# Lipoprotein(a)-associated proteomic signature predicts cardiovascular disease in young adults

**DOI:** 10.1172/JCI204287

**Published:** 2026-04-16

**Authors:** Sascha N. Goonewardena, Shanshan Yao, Tomasz Jurga, Lanyue Zhang, Donald Lloyd-Jones, Dilna Damodaran, Bharat Thyagarajan, David R. Jacobs, Supriya Shore, Eric J. Brandt, Clary Clish, Kahraman Tanriverdi, Jane E. Freedman, Chirag J. Patel, Mark A. Sarzynski, Brian T. Emmer, John T. Wilkins, Ron Do, Vera Bittner, Ravi V. Shah, Marios K. Georgakis, Robert S. Rosenson, Venkatesh L. Murthy

**Affiliations:** 1 Division of Cardiovascular Medicine, Department of Internal Medicine, University of Michigan, Ann Arbor, Michigan, USA.; 2 Division of Cardiovascular Medicine, and; 3 Department of Pharmacy, VA Ann Arbor Health System, Ann Arbor, Michigan, USA.; 4 Institute for Stroke and Dementia Research (ISD), LMU University Hospital, LMU Munich, Munich, Germany.; 5 Department of Preventive Medicine, Northwestern University Feinberg School of Medicine, Chicago, Illinois, USA.; 6 Department of Laboratory Medicine and Pathology, University of Minnesota, Minneapolis, Minnesota, USA.; 7 University of Minnesota School of Public Health, Division of Epidemiology and Community Health, Minneapolis, Minnesota, USA.; 8 Institute for Healthcare Policy and Innovation, University of Michigan, Ann Arbor, Michigan, USA.; 9 Broad Institute of MIT and Harvard, Cambridge, Massachusetts, USA.; 10 Vanderbilt Translational and Clinical Research Center, Cardiovascular Division, Vanderbilt University Medical Center, Nashville, Tennessee, USA.; 11 Department of Biomedical Informatics, Harvard Medical School, Boston, Massachusetts, USA.; 12 Department of Exercise Science, University of South Carolina, Columbia, South Carolina, USA.; 13 Department of Internal Medicine, Division of Hospital Medicine, University of Michigan Medical School, Ann Arbor, Michigan, USA.; 14 Division of Cardiology, Department of Medicine, Northwestern, University Feinberg School of Medicine, Chicago, Illinois, USA.; 15 The Charles Bronfman Institute for Personalized Medicine, Icahn School of Medicine at Mount Sinai, New York, New York, USA.; 16 Department of Medicine, Division of Cardiovascular Disease, University of Alabama at Birmingham, Birmingham, Alabama, USA.; 17 Munich Cluster for Systems Neurology (SyNergy), Munich, Germany.; 18 Program in Medical and Population Genetics and Cardiovascular Disease Initiative, Broad Institute of MIT and Harvard, Cambridge, Massachusetts, USA.; 19 Metabolism and Lipids Program, Zena and Michael A. Wiener Cardiovascular Institute, Mount Sinai Heart, Icahn School of Medicine at Mount Sinai, New York, New York, USA.

**Keywords:** Cardiology, Inflammation, Atherosclerosis, Lipoproteins

## Abstract

**BACKGROUND:**

Elevated lipoprotein(a) [Lp(a)] is associated with a higher risk of atherosclerotic cardiovascular disease (ASCVD). Although Lp(a) is a genetically determined risk factor, the plasma proteomic features associated with Lp(a) and whether they provide information about ASCVD risk beyond Lp(a) concentration are not well characterized.

**OBJECTIVE:**

We sought to identify plasma proteomic features associated with Lp(a) concentration and to evaluate whether an Lp(a)-associated proteomic signature is associated with ASCVD phenotypes in young, healthy adults.

**METHODS:**

In the Coronary Artery Risk Development in Young Adults (CARDIA) study, we measured year 7 Lp(a) and 184 cardiovascular proteins using the Olink proximity extension assay in 3,920 participants without prior coronary heart disease. Lp(a)-associated proteomic signatures were derived using least absolute shrinkage and selection operator (LASSO) regression in a split-sample design and tested for association with coronary artery calcification (CAC), incident coronary heart disease (CHD), and high-sensitivity C-reactive protein (hs-CRP) over 27 years of follow-up. External replication was performed in the UK Biobank (*n =* 37,996).

**RESULTS:**

Lp(a) was associated with CAC (OR 1.23 [1.13–1.34]; *P <* 0.0001) and incident CHD (HR 1.23 [1.07–1.41]; *P =* 0.004). Lp(a) was correlated with proteomic features reflecting immune activation, coagulation, and vascular dysfunction. A quantitative Lp(a)-associated proteomics score was independently associated with incident CAC (standardized β = 0.40, *P <* 0.0001) and hs-CRP (standardized β = 0.11, *P =* 0.00015) after adjustment for Lp(a) concentration. In the UK Biobank, a recalibrated Lp(a)-associated proteomics score was associated with CRP, incident CHD, and all-cause mortality.

**CONCLUSIONS:**

In young adults, Lp(a) was associated with distinct proteomic features that independently predicted ASCVD phenotypes beyond Lp(a) concentration, generating hypotheses regarding biological pathways linked to Lp(a)-related cardiovascular risk.

**FUNDING:**

VA MERIT grant (1I01CX002560); Taubman Medical Research Institute (Wolfe Scholarship); National Institute of Diabetes, Digestive, and Kidney Diseases (NIDDK), NIH (U01DK123013-03); National Institute on Aging (NIA), NIH (R01AG059729); National Heart, Lung and Blood Institute (NHLBI), NIH (R01HL136685); American Heart Association Strategically Focused Research Network grant in Cardiometabolic Disease (funded proteomics in CARDIA); NIH (K23MD017253 and R01HL167733); Blue Cross Blue Shield of Michigan Foundation; A. Alfred Taubman Medical Research Institute; National Institute of Nursing Research (R01NR019628); National Institute of General Medical Sciences (NIGMS), NIH (R35-GM124836). The CARDIA study was conducted and supported by the NHLBI in collaboration with the University of Alabama at Birmingham (75N92023D00002 and 75N92023D00005), Northwestern University (75N92023D00004), University of Minnesota (75N92023D00006), and the Kaiser Foundation Research Institute (75N92023D00003).

**ROLE OF FUNDING SOURCE:**

The funders had no role in the design of the study; in the collection, analyses, or interpretation of data; in the writing of the manuscript; or in the decision to publish the results.

## Abstract 2

Lp(a)-associated proteomic signatures, reflecting immune and vascular pathways, predict ASCVD risk and mortality independently of Lp(a) concentration, highlighting biological mechanisms beyond simple mass measurements.

## Graphical abstract

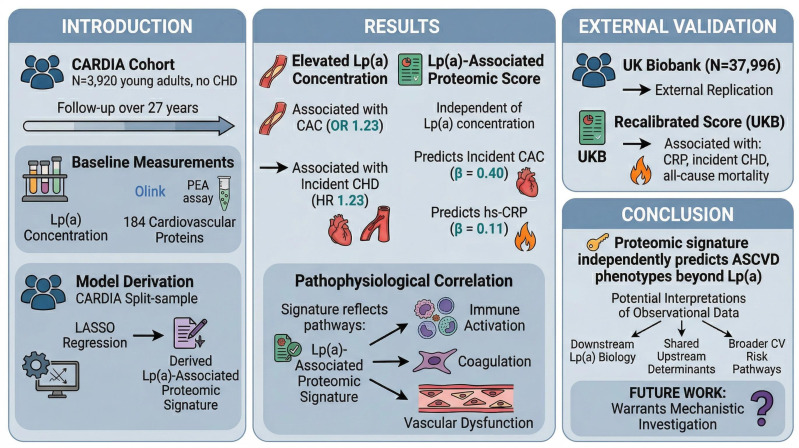

## Introduction

Despite major therapeutic advances, atherosclerotic cardiovascular disease (ASCVD) remains the leading cause of morbidity and mortality worldwide (1). This is due to a high prevalence of traditional risk factors, including a lack of heart-healthy dietary practices and an aging population (2). However, even after accounting for traditional risk factors, substantial ASCVD risk remains. Part of this risk is mediated by elevated levels of lipoprotein(a) [Lp(a)]. Elevated concentrations of Lp(a) increase the risk for ASCVD events in persons with or without coronary heart disease (CHD) (3–5). Observational cohort studies, post hoc analyses of clinical trials, and Mendelian randomization studies suggest an independent, likely causal relationship between elevated Lp(a) levels and ASCVD risk (6–9). Although there are currently no approved pharmacological therapies that directly target elevated Lp(a), several therapies are in development that reduce apolipoprotein(a) production by up to 80%–99% (10).

Mechanistically, Lp(a) promotes ASCVD through multiple pathways, including atherogenesis, thrombosis, and inflammation (11). Genetic studies suggest that, on a per-particle basis, Lp(a) is associated with greater atherosclerosis than LDL, indicating that Lp(a) has additional atherogenic properties beyond LDL (12). Given that a major structural component of Lp(a) is encoded by a gene (*LPA*) that was duplicated from the plasminogen gene, it has been hypothesized that altering fibrinolysis could influence some of the effects of Lp(a) on thrombosis (13–15). However, several studies in murine models and human cohorts have failed to demonstrate a clear effect of Lp(a) on fibrinolysis or venous thromboembolic disease (16). Regarding inflammation, several basic and observational studies have demonstrated a relationship between Lp(a) and the immune system (17, 18). In asymptomatic middle-aged participants in the Multi-Ethnic Study of Atherosclerosis (MESA), Lp(a) ASCVD risk and all-cause mortality were only observed in combination with an elevated high-sensitivity C-reactive protein (hs-CRP) (>2 mg/L) (19). More recently, a risk factor and context-dependent relationship between Lp(a) and inflammation has emerged (20). A study by Arnold and colleagues found that high levels of Lp(a) were associated with an increased risk of CHD events, regardless of the level of hs-CRP in individuals without CHD (21). However, in individuals with established CHD, Lp(a) was only associated with recurrent CHD events in those with high hs-CRP levels. However, the relationship with Lp(a) and hs-CRP is complex, and not all studies have found robust relationships between Lp(a) and hs-CRP (22–23). Functional evidence linking Lp(a) to inflammation has been revealed in experimental models, and clinical studies have demonstrated that Lp(a) is the primary lipoprotein carrier of phosphocholine-containing oxidized phospholipids (OxPL), a damage-associated molecular pattern (DAMP) that can be recognized by pattern recognition receptors (PRRs) on immune and nonimmune cells (24–25). Lp(a) appears to influence multiple immune pathways in a context- and individual-specific manner, potentially accelerating ASCVD.

Although Lp(a) is associated with ASCVD, the molecular features and pathways linking elevated Lp(a) to distinct ASCVD phenotypes remain incompletely understood. We first examined associations between Lp(a) measured in young, healthy adults and the development of subclinical and clinical ASCVD events over 25 years of follow-up in the Coronary Artery Risk Development in Young Adults (CARDIA) study. To generate hypotheses regarding candidate pathways associated with Lp(a)-related cardiovascular risk, we leveraged proteomics profiling in combination with longitudinal phenotyping of young, healthy adults in the CARDIA study. Using a data-driven machine-learning framework, we integrated plasma proteins measured in 2,303 participants coincident with Lp(a) levels to construct Lp(a)-associated proteomic signatures. We then examined whether these signatures capture pathway-specific associations between Lp(a) and subclinical atherosclerosis (coronary artery calcification [CAC])), systemic inflammation (CRP), and incident clinical events (coronary heart disease, mortality). External replication with recalibration in over 37,000 UK Biobank participants supported the biological plausibility of the key CARDIA findings. This hypothesis-generating approach integrates Lp(a)-associated proteomic signatures with long-term ASCVD outcomes in population-based cohorts, providing biological associations that may inform future mechanistic investigations of how Lp(a) relates to atherosclerotic disease across its natural history.

## Results

### Characteristics of the CARDIA cohort.

Lp(a) was measured at year 7 (Y7) in 3,920 participants without prior CHD. The demographic and laboratory characteristics of this group are shown in Table 1. The distribution of age and sex was similar across Lp(a) strata (Table 1 and Supplemental Figure 1; supplemental material available online with this article; https://doi.org/10.1172/JCI204287DS1).

### Lp(a) is associated with clinical markers of inflammation, atherosclerosis, and CHD events in young, healthy adults.

**Figure 1:**
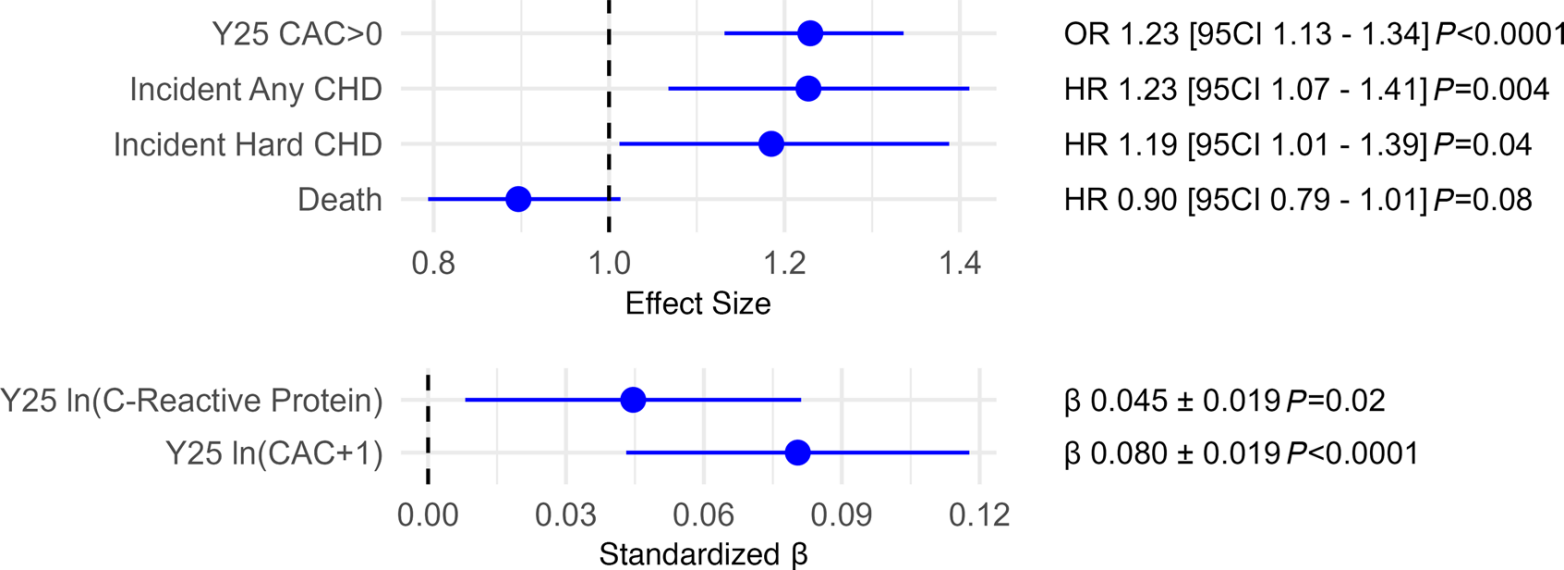
Lp(a) levels in young, healthy adults are associated with inflammatory and atherogenic phenotypes as well as CHD. Forest plot of standardized regression coefficients from models relating Y7 Lp(a) (as a continuous variable) to CAC (binary and natural log-transformed + 1), hs-CRP, and CHD endpoints (outcome variable). Logistic regression was used for binary outcomes (CAC presence), linear regression for continuous outcomes (hs-CRP, ln[CAC+1]), and Cox proportional hazards regression for time-to-event outcomes (CHD). Models were adjusted for age, sex, and race.

To understand how Lp(a) levels relate to the risk of subclinical and clinical ASCVD, we assessed the data on CARDIA participants with a median follow-up of 27.1 years. We quantified the risk of ASCVD (and associated atherogenic and inflammatory phenotypes) across the entire range of observed Lp(a) concentrations as well as through Lp(a) quintiles. With respect to atherogenesis and inflammation, Lp(a) was linked to CAC and hs-CRP (Figure 1 and Supplemental Figure 2). In the model adjusted for age, sex, and race, Lp(a) as a continuous variable was associated with incident hard and any CHD (Figure 1). The incidence of any and hard CHD was 1.82 per 1,000 person-years and 1.43 per 1,000 person-years in the highest quintile of Lp(a); in contrast, the incidence of any and hard CHD was 1.03 per 1,000 person-years and 0.93 per 1,000 person-years in the lowest quintile of Lp(a) (Supplemental Figure 3). To contextualize the risk gradient across quintiles of Lp(a), we also conducted similar quintile analyses for LDL cholesterol (LDL-C) and hs-CRP and how these related to CHD outcomes. With respect to any and hard CHD events, for the highest quintile of LDL-C, the incidence was 3.13 per 1,000 person-years and 2.37 per 1,000 person-years, respectively (Supplemental Figure 3), as compared with the lowest quintile of LDL-C. For hs-CRP, those in the highest quintile had incidence rates of any and hard CHD events of 1.75 per 1,000 person-years and 1.5 per 1,000 person-years, respectively, more closely resembling those of the highest quintile of Lp(a) than of LDL-C. In young, healthy adults, Lp(a) levels were linked not only to CHD endpoints but also to atherogenic and inflammatory phenotypes.

### Lp(a) levels in young, healthy adults are linked to specific proteomic features.

**Figure 2:**
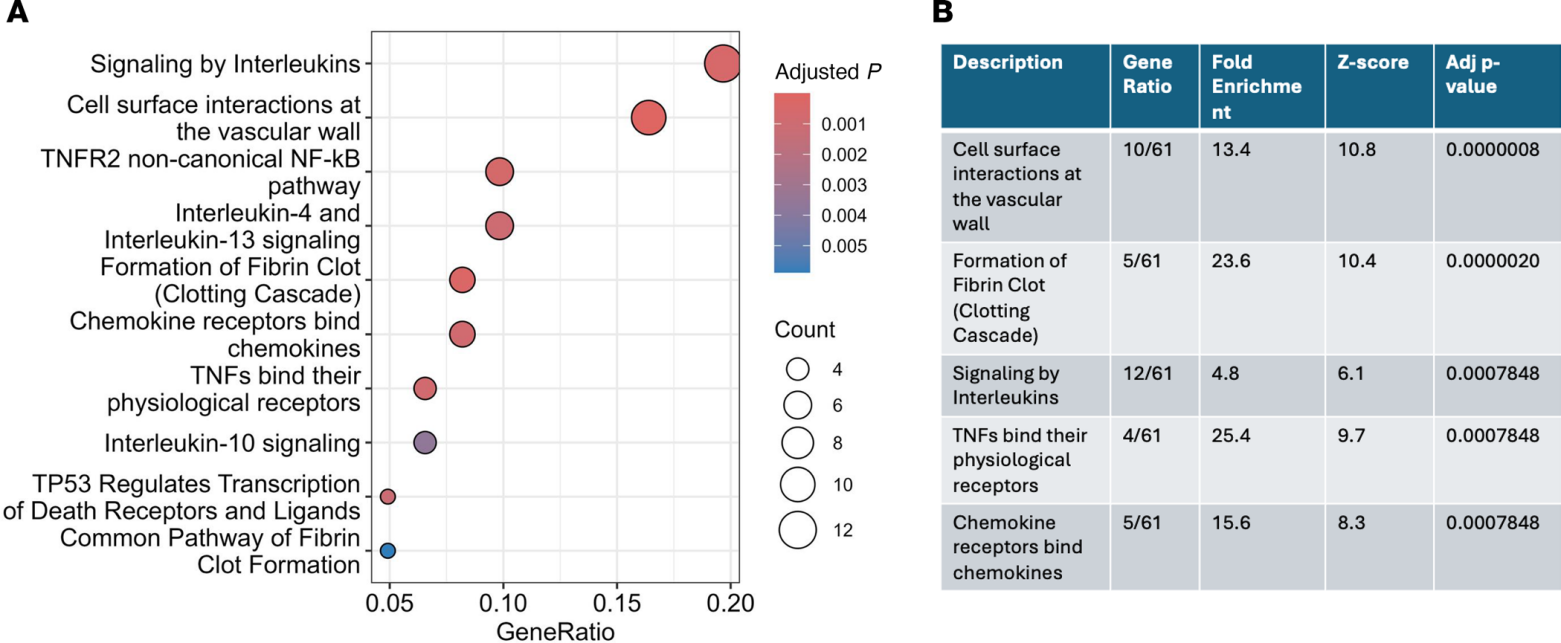
Lp(a) as a predictor of proteomic features. Sixty-two proteomic features were selected using single regression analyses, and pathway analyses were performed. (**A**) Dot plot of pathway topology results with adjusted *P* values and counts. The size of the dot reflects the counts, and the color captures the adjusted *P* value. (**B**) Top 5 significant pathways from the 62 mapped proteomic features. Proteins were selected by linear regression of Lp(a) on individual proteins (*P <* 0.05); pathway enrichment was assessed using the REACTOME database with Benjamini-Hochberg correction.

Although Lp(a) affects ASCVD through various mechanisms, there is considerable heterogeneity in how these pathological processes contribute to the distinct clinical presentations of ASCVD. To better understand the biological pathways that may link Lp(a) and ASCVD, we measured circulating proteins in young adulthood (Y7). We examined how Lp(a) levels related to these proteomic features. The characteristics of this CARDIA subcohort [having Y7 Lp(a) and proteomics; *n =* 2,303] are shown in Supplemental Table 1. The results of linear, logistic, and Cox models are presented in Figure 2 and the Supporting Data Values file. From the single-variable regression analyses using Y7 Lp(a) levels as the predictor, we identified 62 unique proteins with robust associations. These proteins were mapped to biological pathways using the REACTOME database. Figure 2 summarizes the standardized regression coefficients across pathways prioritized in the analysis. Pathway enrichment analysis revealed substantial involvement of processes such as signaling by interleukins, cell-surface interactions at the vascular wall, and formation of fibrin clot, suggesting that these biological mechanisms are linked to Lp(a) concentrations.

### Derivation of an Lp(a)-associated proteomics score in young, healthy adults.

**Figure 3:**
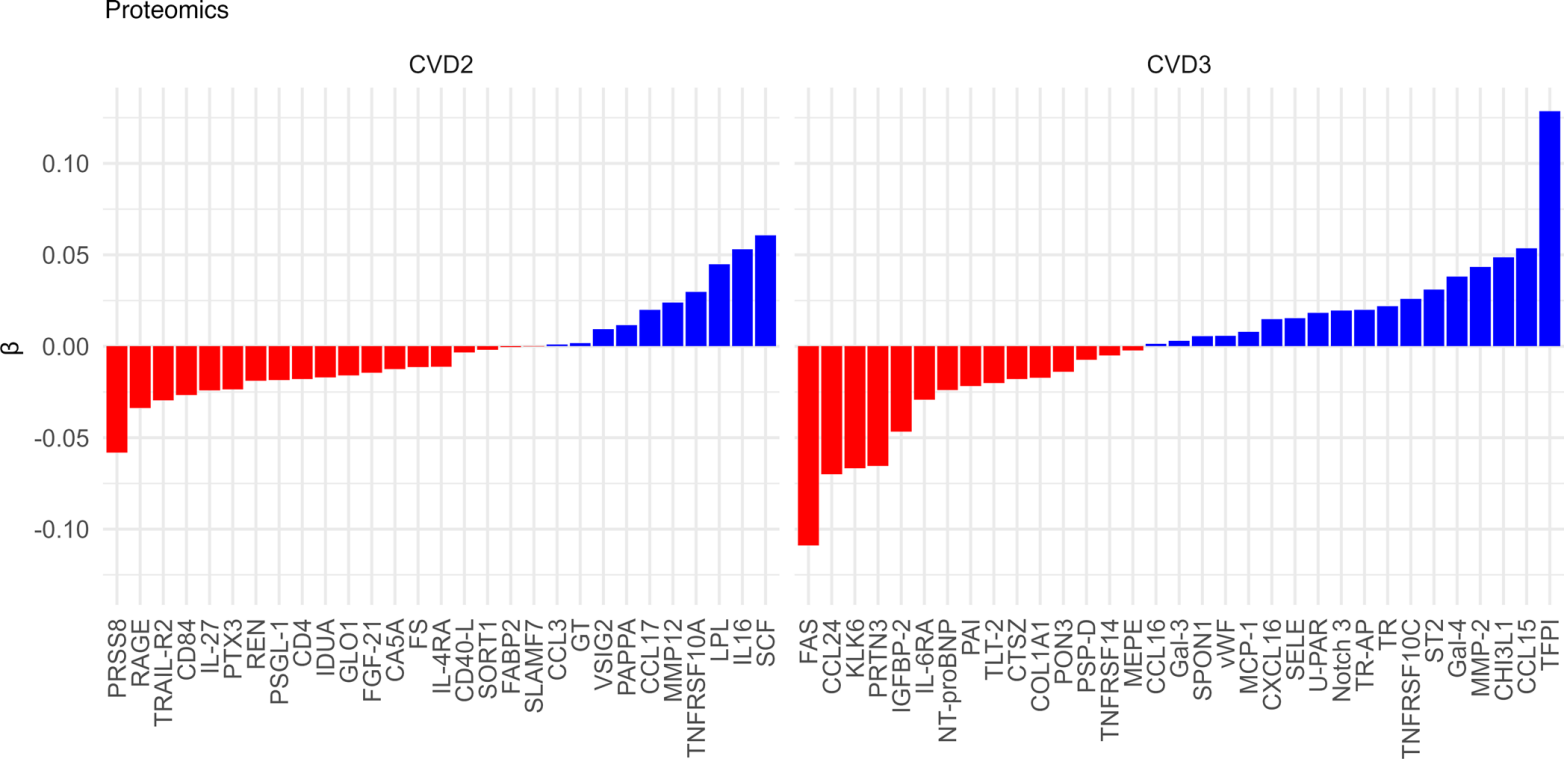
LASSO derived Lp(a)-associated proteomics scores and individual proteomic feature contributions. Standardized regression coefficients for proteins are depicted as bars, with the length of the bar proportionate to the weight, red color for negative weights, and blue color for positive weights. Bars are grouped according to the proteomics platform used (Olink CVD II: 92 proteins related to CVD pathways; Olink CVD III: 92 proteins related to CVD pathways, with partial overlap between panels). Protein weights were derived by LASSO penalized regression (α = 1) with 5-fold cross-validation in the 70% derivation subset.

Using least absolute shrinkage and selection operator (LASSO) regression analysis, we derived Lp(a) quantitative scores composed of the most strongly associated proteins (Figure 3). Not surprisingly, in the validation cohort (*n =* 691, 30% holdout), the proteomics scores showed a notable correlation with Y7 Lp(a) levels from which they were derived, with a Pearson correlation coefficient of 0.32 [out-of-sample *R²* = 0.102 for continuous Lp(a) in nmol/L; *R²* = 0.137 for rank-normalized Lp(a), *r* = 0.37] (Supplemental Figure 4). When examining the specific parts of the proteomics score that influenced the association with Y7 Lp(a), several observations are worth noting. Among specific proteins, some of the most substantial contributions (both positive and negative) to the Lp(a)-associated proteomics score included TFPI, MMP2, RAGE, SCF, LPL, CCL24, and NT-proBNP, all of which have been linked to ASCVD (Figure 3). These findings suggest that metabolic derangements, vascular dysfunction, and immune activation contribute to the molecular mechanisms linked to Lp(a),providing hypothesis-generating insights into biological pathways associated with Lp(a) and ASCVD.

### The Lp(a)-associated proteomics score is independently associated with ASCVD phenotypes after adjustment for Lp(a) concentration.

**Figure 4:**
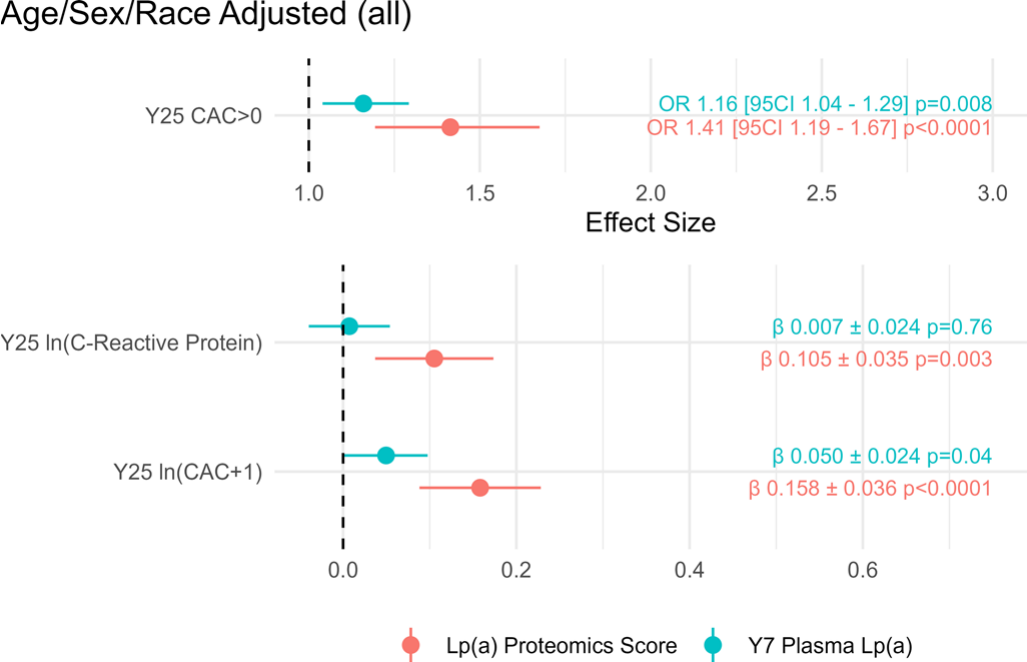
Association of Y7 plasma Lp(a) and Lp(a)-associated proteomics score with year 25 subclinical atherosclerosis and inflammation in the CARDIA study. Adjusted associations of Y7 plasma Lp(a) (teal) and Lp(a)-associated proteomics score (coral) with Y25 CAC presence (CAC >0, top), ln(CRP) (middle), and ln(CAC+1) (bottom). When both were included in the same models, the Lp(a)-associated proteomics score retained independent associations with all 3 outcomes, whereas the Lp(a) concentration did not reach significance. β, standardized β coefficient. Logistic regression was used for CAC presence (OR), and linear regression for ln(CRP) and ln(CAC+1) (standardized β). Models were adjusted for age, sex, and race. ln, natural logarithm.

Given the complex relationships among Lp(a), pathogenic pathways, and ASCVD phenotypes, we used the quantitative Lp(a)-associated proteomics score to examine how the proteomic signature correlates with clinical markers of inflammation (hs-CRP), atherosclerosis (CAC), and CHD outcomes. Although the Lp(a)-associated proteomics score correlated with measured Lp(a), the correlation was moderate (Supplemental Figure 4), suggesting that the score may capture biological variation associated with ASCVD that is not fully explained by the Lp(a) concentration. In brief, we used a statistical approach in which atherogenic (CAC), inflammatory (hs-CRP), and ASCVD events were modeled using the Lp(a)-associated proteomics score (Figure 4). Because the CARDIA-omics subcohort had fewer participants (*n =* 2,303), the strength of the associations between Lp(a) and clinical and subclinical ASCVD endpoints was attenuated compared with the larger cohort (*n =* 3,920). In this young, healthy subcohort, measured Lp(a) alone showed minimal associations with ASCVD endpoints compared with the Lp(a)-omics scores. For example, when comparing the Y7 Lp(a)-associated proteomics score with Lp(a) in the adjusted model, Lp(a) had minimal independent associations with ASCVD phenotypes. When both the Lp(a)-associated proteomics score and Lp(a) concentration were included in the same adjusted models, the proteomics score remained independently associated with Y25 incident CAC (standardized β = 0.40, *P <* 0.0001) and hs-CRP (standardized β = 0.11, *P =* 0.00015), whereas the Lp(a) concentration did not reach significance for these endpoints (Figure 4 and Supporting Data Values file). These findings indicate that the proteomics score is associated with ASCVD phenotypes independently of Lp(a) concentration, although whether this reflects downstream Lp(a) biology, shared upstream determinants, or correlated cardiovascular risk pathways cannot be determined from these data.

To further explore and validate the associations between the Lp(a)-associated proteomics score and ASCVD phenotypes, we examined how the Lp(a)-associated proteomics score associates with ASCVD phenotypes in an external cohort. We identified 37,996 individuals from the UK Biobank who were free of CHD at baseline and had Lp(a) and proteomics data (Olink Explore 1536) and phenotypes related to survival and ASCVD (Supplemental Table 2). Four outcomes were assessed over a median follow-up of 13.6 years, including incident hard CHD, incident any CHD, all-cause death, and baseline CRP levels. Cox proportional hazards models were used for time-to-event outcomes and linear regression for CRP. Because of cohort-specific differences in baseline characteristics and proteomics platforms, we performed external replication with recalibration of the Lp(a)-associated proteomics score using the same set of proteins linked to Lp(a) in the CARDIA cohort. The Lp(a)-associated proteomics score was strongly correlated with baseline CRP levels (fully adjusted *P =* 1.42 × 10^–54^), incident any CHD (fully adjusted *P =* 0.001), and all-cause mortality (fully adjusted *P <* 0.01). Of the 62 proteins selected by LASSO in CARDIA, 61 were available on the Olink Explore 1536 platform in the UK Biobank (matrix metalloproteinase-2 [MMP-2] was unavailable). The score was recalibrated by reweighting the original CARDIA-derived LASSO coefficients in the UK Biobank derivation subset to account for platform differences rather than re-running LASSO on UK Biobank data.

Formal model comparisons showed that Lp(a) contributed modestly to CAC prediction beyond demographic and anthropometric factors (likelihood ratio test [LRT] *P =* 0.040 for the age/sex/race model), although improvements were attenuated in fully adjusted models. We cannot exclude the possibility that the modest incremental discrimination reflects sample size limitations in the proteomics subcohort (Supplemental Table 5). The association between Lp(a) and CAC was consistent across hs-CRP tertiles, with no significant interaction (*P =* 0.88 for CAC presence, *P =* 0.39 for CAC severity), suggesting that the Lp(a)-associated findings were not driven solely by inflammatory status (Supplemental Table 6). Race-stratified analyses demonstrated directionally consistent associations between Lp(a) and CAC in both Black and White participants, with no significant effect modification by race for CAC endpoints (interaction *P =* 0.73 for CAC presence, *P =* 0.78 for CAC severity). The Lp(a)-CVD association was nominally stronger in White participants (HR 1.003 per 1 nmol/L Lp(a), *P =* 0.030) than in Black participants (HR 1.0003 per 1 nmol/L Lp(a), *P =* 0.78), although the formal interaction was not statistically significant (*P =* 0.12). A significant Lp(a)-race interaction was observed for hs-CRP (interaction *P =* 0.021), with a positive Lp(a)-CRP association in Black participants that was absent for White participants, which may reflect known racial differences in Lp(a) biology and inflammatory pathway activation (Supplemental Table 7).

## Discussion

In this study of young, healthy adults enrolled in the CARDIA study, we explored the relationships between Lp(a) levels and proteomic features and how these relate to the development of subclinical and clinical ASCVD phenotypes. Our key findings were: (a) Lp(a) levels in a young, healthy cohort were linked to accelerated atherogenesis, inflammation, and early CHD outcomes; (b) Lp(a) levels correlated with specific proteomic signatures; (c) an Lp(a)-associated proteomics score was associated with increased inflammation (hs-CRP), atherosclerosis (CAC), and ASCVD phenotypes; (d) when both the Lp(a)-associated proteomics score and Lp(a) concentration were included in the same models, the proteomics score retained independent associations with ASCVD phenotypes; and, finally, (e) the Lp(a)-associated proteomics score correlated with CHD and all-cause mortality in a large external replication cohort. These findings connect Lp(a) with specific proteomic features and demonstrate that an Lp(a)-associated proteomics score predicts subclinical and clinical ASCVD phenotypes over extended follow-up periods, generating hypotheses regarding biological pathways that may be associated with Lp(a)-related ASCVD.

Building on previous findings from higher-risk populations, we found that in young, healthy individuals, Lp(a) was associated with subclinical ASCVD phenotypes and CHD risk. Specifically, Lp(a) was linked not only to incident CHD but also to atherogenic phenotypes (e.g., Y25 CAC) and markers of increased inflammation (e.g., hs-CRP at Y25). A study of middle-aged participants in the UK Biobank further showed that higher Lp(a) levels were linked to an increased risk of ASCVD events (26). This difference in risk [between participants with high and low Lp(a) levels] was more evident in individuals with no previous history of ASCVD. Additionally, a study involving participants from the UK Biobank, along with further analyses from the FOURIER and SAVOR-TIMI 53 trials, showed that elevated Lp(a) levels were associated with a higher ASCVD risk in both primary and secondary prevention groups, regardless of baseline hs-CRP (27). One distinctive feature of our study is the analysis of a younger patient population with fewer cardiovascular risks, i.e., lower rates of hypertension, diabetes, and baseline LDL-C, compared with MESA, Atherosclerosis Risk in Communities (ARIC), and the UK Biobank participants (19, 28–30). In higher-risk populations, LDL-C and hs-CRP have been linked to ASCVD phenotypes. Consistent with these findings, we also observed that LDL-C and hs-CRP were associated with CHD, and Lp(a) risk aligned more closely with hs-CRP in terms of the extent of ASCVD risk. These findings have substantial implications, emphasizing that Lp(a) is less variable than other ASCVD risk markers. For instance, markers like LDL-C respond to lifestyle changes and medication, whereas Lp(a) levels remain largely stable (31). Interestingly, possibly because of the lower ASCVD risk in the CARDIA cohort, we observed a trend toward increased mortality in the lowest quintile of Lp(a), which did not align with ASCVD events. The total number of deaths was low, and neither the magnitude nor the significance was affected by Lp(a) levels. Further research is needed to clarify this unclear finding, which might be due to competing risks or simply a chance occurrence. Importantly, this result was not seen in the external replication analyses from the UK Biobank. Overall, our findings extend the ASCVD Lp(a) research from other observational groups to a younger, lower-risk population, connecting Lp(a) to markers of inflammation, atherogenesis, and ASCVD phenotypes.

Clinical trials with RNA-based therapeutics that reduce Lp(a) levels by 80%–99% are ongoing (10). They will directly test the “Lp(a) hypothesis” that profound reductions in Lp(a) levels will reduce the risk of ASCVD. To complement these clinical outcomes trials, an understanding of the underlying mechanisms by which Lp(a) accelerates ASCVD and how patient-specific risk factors influence these mechanisms will not only help identify the patients most likely to benefit from these therapies but also expand our knowledge of ASCVD itself. Our hypothesis-generating proteomics findings corroborate known associations and identify what we believe to be previously unreported statistical links between Lp(a) and pathways associated with ASCVD. Among the correlates of Lp(a) in our single regression analyses, we identified several markers of vascular dysfunction (PAR1, PSGL1) and inflammation (IL-6–RA, MCP1), all of which have been associated with ASCVD (32–36). From a pathway perspective, the immune and vascular pathways (e.g., signaling by interleukins, cell-surface interactions at the vascular wall) implicated in the proteomics analyses have clear links to ASCVD (37). As alluded to earlier, proteomics approaches not only offer insights into quantitative, patient-specific risk but also reveal potential mechanisms that contribute to ASCVD heterogeneity.

Using proteomics features, we developed a quantitative Lp(a)-associated proteomics score and examined its relationship to ASCVD phenotypes. ASCVD heterogeneity reflects the complex interplay of processes that contribute to its progression. Lp(a) has been associated with atherogenesis, thrombosis, and inflammation in prior experimental and observational studies, each of which has been implicated in ASCVD. Evidence of this is seen in our data, in which Lp(a) correlates with pathway-specific molecular features and clinical markers of inflammation (hs-CRP) and atherogenesis (CAC). The individual Lp(a) proteomic features showed minimal associations with ASCVD phenotypes. However, the machine-learning quantitative scores, a composite of the individual proteomic features, were associated with ASCVD phenotypes and retained independent associations when Lp(a) concentration was included in the same models.

The observational nature of our study precludes strict determinations of causality with respect to Lp(a) pathobiology, and we wish to present 3 interpretations of our findings with equal weight. First, the identified proteomic associations may reflect proteins that mediate the downstream effects of Lp(a) on cardiovascular risk, in which case the score would represent a readout of Lp(a) biology more proximal to disease than Lp(a) concentration alone. Second, the score and Lp(a) may share common upstream genetic or metabolic determinants, such that both are markers of the same underlying pathobiology without a direct Lp(a)-to-protein causal chain. Third, the score may capture broader cardiovascular risk biology that is statistically correlated with Lp(a) but not biologically downstream of it. The moderate correlation between the score and Lp(a) concentration (*r* = 0.32) and the observation that the score predicts ASCVD more strongly than Lp(a) itself are consistent with each of these interpretations. Distinguishing among them will require future studies using Mendelian randomization with protein quantitative trait loci, or experimental validation in model systems. We view our findings as hypothesis generating and intended to inform the design of such mechanistic investigations.

The moderate correlation between the Lp(a)-associated proteomics score and Lp(a) concentration (*r* < 0.4) warrants discussion. This correlation is expected, given the LASSO regularization approach, which selects proteins that contribute information beyond Lp(a) levels alone. However, the observation that the score associates more strongly with cardiovascular outcomes than Lp(a) itself raises important interpretive questions. This pattern could indicate that the score captures downstream biological effects of Lp(a) that are more proximal to disease pathogenesis than Lp(a) concentration. Alternatively, the score may capture pathways that share common upstream determinants with Lp(a), or cardiovascular risk biology that is statistically but not causally linked to Lp(a). Distinguishing among these possibilities will require causal inference methods such as Mendelian randomization with protein quantitative trait loci, which is beyond the scope of the present study.

We acknowledge that the Olink CVD panels used in this study are enriched for proteins involved in inflammation, lipid metabolism, and cardiovascular biology. This targeted design means that pathway analyses will inherently identify cardiovascular system–related processes, limiting the novelty of pathway-level findings. However, the panel composition did not predetermine which specific proteins were associated with Lp(a), and not all cardiovascular pathways represented on the panel were enriched among Lp(a)-associated proteins. Future studies using unbiased discovery proteomics platforms would strengthen the biological interpretation of these findings and may identify additional Lp(a)-associated pathways not represented on targeted cardiovascular panels.

The absence of a statistically significant Lp(a)-CVD association in Black participants (HR 1.0003 per 1 nmol/L Lp(a), *P =* 0.78) despite higher mean Lp(a) levels (38) may reflect several nonmutually exclusive factors: differential Apo(a) isoform size distributions across racial groups, Olink proximity extension assay (PEA) nonlinearity at higher analyte concentrations (39), competing mortality, and differential statistical power in race-stratified subgroups. Notably, the Lp(a)-CAC association was directionally consistent across both racial groups (interaction *P =* 0.73), suggesting that the discrepancy may be specific to clinical CVD events. Future studies in larger, racially diverse cohorts with isoform-specific Lp(a) measurements will be needed to characterize these relationships.

### Study limitations.

Several limitations of our study warrant consideration. First, although our clinical assay for Lp(a) concentration involved the use of an isoform-insensitive immunoassay, we did not have information on oxidized phospholipid content that may more precisely link proteomic features to distinct Lp(a) properties (40). Additionally, our design was a split set, as the ASCVD risk factor distribution (e.g., age and diabetes) and protein coverage were unique to this cohort. Although the uniqueness of our cohort captured an understudied early time point in Lp(a)-driven ASCVD risk, we performed external replication with recalibration of these findings in the UK Biobank. While external replication in the UK Biobank largely confirmed the CARDIA findings, the younger age of the CARDIA cohort and low event rates among younger UK Biobank participants limited our ability to evaluate associations specifically in early adulthood. With respect to LDL-C measurements, it is known that Lp(a)-C is included in standard determinants of LDL-C. In our analyses presented here, we elected not to correct for Lp(a)-C in our LDL-C measurements. A recent study has explored the importance of this known phenomenon, and within the context of the populations examined, the effect of correcting LDL-C for Lp(a)-C was minimal (41). Furthermore, the observational design of our study limits our ability to establish causality regarding Lp(a) pathobiology; however, our results are hypothesis-generating and can guide future mechanistic and validation studies. Related to this limitation, we recognize that residual confounding may persist in observational studies such as ours, despite careful covariate adjustment and external replication. Finally, we recognize that Lp(a) testing is currently underused in clinical practice, and increasing access to direct Lp(a) measurement should remain a top priority. The proteomics score described here is not intended to replace Lp(a) testing but to provide additional information and biological insights that may help improve our understanding of ASCVD risk assessment.

Direct assessment of renal function was not available at the Y7 examination. However, CARDIA participants were young adults (mean age, 32 years), and analysis of available creatinine-derived estimated glomerular filtration rate (eGFR) data confirmed that clinically meaningful chronic kidney disease (CKD) was exceedingly rare in this cohort: only 0.79% at baseline and 0.45% at Y10 had CKD stage 3 or higher, and over 99% of the cohort had preserved renal function. Formal sensitivity analyses incorporating Y10 creatinine-derived eGFR (CKD–Epidemiology Collaboration [EPI] 2009; mean 98.9 ± 16.7 mL/min/1.73 m²) into fully adjusted models demonstrated negligible attenuation of effect estimates across all primary endpoints (all changes <5%), confirming that renal function variation did not confound the observed associations. The use of Y10 eGFRs, measured approximately 3 years after proteomics profiling, represents an imperfect temporal match; however, in a young adult cohort with minimal kidney disease progression, this offset is not likely to materially affect the results. These findings are consistent with published data from this cohort and indicate that the variability in renal function was insufficient to produce meaningful confounding by differential renal clearance of measured proteins (Supplemental Table 8).

Several limitations of our external replication analyses merit discussion. The UK Biobank analyses required recalibration of the proteomics score due to differences in the Olink assay panels and population characteristics, which means the analysis does not test the direct transportability of the original CARDIA-derived score weights. However, this approach does test whether the underlying biological relationships between Lp(a) and the identified proteomic signatures are reproducible in an independent population measured on a different platform. Additionally, the UK Biobank cohort is substantially older and less racially diverse than the CARDIA cohort, which limits our ability to validate findings specifically in young adults. Future studies in independent young adult cohorts with identical proteomics platforms will be essential to establish the transportability of the Lp(a)-associated proteomics score as derived.

The Olink PEAplatform, while offering high sensitivity and specificity for targeted protein measurement, represents an affinity-based approach with inherent limitations. Cross-reactivity, epitope accessibility, and protein complex formation may influence measured protein levels (42). Future studies using complementary proteomics technologies, including mass spectrometry–based approaches, will strengthen the biological interpretation of our findings.

### Conclusions.

In summary, we derived an Lp(a)-associated proteomics score in young, healthy adults that independently predicted subclinical and clinical ASCVD phenotypes over 27 years of follow-up, with replication in the UK Biobank. Whether the score reflects downstream Lp(a) biology, shared upstream determinants, or correlated cardiovascular risk pathways remains to be determined. Regardless of the precise biological mechanism, a proteomics score that predicts ASCVD in young adults represents a potentially useful tool for understanding cardiovascular risk and generates hypotheses for future mechanistic and causal inference studies to elucidate the pathobiology of Lp(a)-related cardiovascular risk.

## Methods

### Sex as a biological variable.

This study analyzed data from NHLBI-funded observational cohort studies that enrolled both female and male participants. Sex was prespecified as a biological variable in the study design. Analyses were conducted of the overall cohort and, where appropriate, stratified by sex or adjusted for sex as a covariate to account for potential biological differences. Interaction testing was performed to evaluate whether observed associations differed by sex. Because the cohorts include representation of both sexes and the underlying cardiometabolic, and inflammatory pathways under study are operative in both females and males, the findings are expected to be broadly relevant to both sexes.

### Study participants and Lp(a) measurements.

The CARDIA study is a prospective, observational cohort study of 5,115 participants (White and Black, aged 18–30 years) recruited in 1985 and 1986 from Birmingham, Alabama; Chicago, Illinois; Minneapolis, Minnesota; and Oakland, California. At inception, 5,115 participants, aged 18–30 years, were enrolled, with a balanced distribution by age, education status, and self-reported sex and race. Race and ethnicity were self-reported by participants at enrollment using investigator-defined categories (Black and White) in accordance with the study design. CARDIA participants underwent in-person examinations at baseline (Y0) and at Y2, Y5, Y7, Y10, Y15, Y20, Y25, Y30, and Y35. Retention rates among surviving participants at each in-person examination were 91%, 86%, 81%, 79%, 74%, 72%, 72%, 71%, and 67% (during the COVID-19 pandemic), respectively. A total of 1,156 individuals were excluded due to missing Y7 Lp(a) data; 36 were excluded for missing key covariates (age, sex, race, total cholesterol, HDL-C, LDL-C, triglycerides, diabetes status, systolic and diastolic blood pressure (BP), use of antihypertensive medications, and smoking status at Y7); and 2 for incident CHD before Y7 resulting in an analysis sample of 3,920 [full cohort with Lp(a) measured at Y7] and *n =* 2,303 [subcohort with Lp(a) and proteomics at Y7]. Plasma Lp(a) analyses were performed using an ELISA method that was insensitive to Apo[a] size heterogeneity (38).

### Proteomics profiling.

Plasma proteomics profiling was performed using the Olink PEA platform (Olink CVD II and CVD III panels) targeting 184 CVD-related proteins on Y7 samples in CARDIA (43). This PEA technique generates amplification cycle numbers that are normalized (Olink NPX Manager Software, version 3.1.1.399) to protein expression levels (NPX) (arbitrary units on a log_2_ scale).

### CARDIA outcomes definitions.

Contact was maintained with participants via telephone, mail, or email every 6 months, with annual interim medical history ascertainment. Approximately 90% of the surviving cohort members were directly contacted, and follow-up for vital status was virtually completed through related contacts and intermittent searches of the National Death Index. For this study, we evaluated clinical events (incident hard CHD, any CHD, and death from any cause as adjudicated per the CARDIA protocol), and subclinical coronary atherosclerosis as measured by Agatston CAC scores on CT at Y15, Y20, and Y25 (44). CAC scores were treated either continuously (after transformation as the natural log transformation of the score plus 1) or dichotomized (as zero or non-zero). hs-CRP was measured at Y7, Y15, Y20, and Y25. Hard CHD was defined as nonfatal myocardial infarction (MI), nonfatal, non-MI acute coronary syndrome, definite fatal MI, and definite or possible fatal CHD. For “any CHD,” coronary revascularization was added to the hard CHD composite of outcome. Time-to-event analyses were defined from the Y7 examination date, with a median follow-up of 27.1 years.

Additional details can be found in the Supplemental Methods.

### Statistics.

We compared continuous and categorical variables across groups using Wilcoxon rank-sum and χ^2^ tests, respectively. We graphically evaluated the distributions and elected to use a natural log transformation of Lp(a) to improve normality (Supplemental Figure 5). Additionally, we converted Lp(a) to a binary variable by dichotomization at 150 nmol/L and used quintiles for further analyses. Quintile and threshold-based analyses included the same participants, with the threshold analysis representing a clinically motivated dichotomization overlapping with (but not identical to) the highest quintile. The high Lp(a) concentration was defined as ≥150 nmol/L (70 mg/dL) or greater based on the inclusion criteria of the Lp(a)HORIZON clinical trial. We used linear, logistic, Cox, and Poisson regression for continuous, binary, time-to-event, and event-rate outcomes, respectively. We scaled key continuous predictors and outcomes to unit variance and a mean of zero to present standardized β coefficients. These regressions were performed without adjustment (unadjusted), adjusted for age/sex/race, or fully adjusted (for age, sex, race, total cholesterol, HDL-C, LDL-C, triglycerides, diabetes status, systolic and diastolic BP, use of antihypertensive medications, and smoking status [all at Y7]). Event rates for incident hard and any CHD, as well as all-cause mortality, across quintiles of Lp(a), LDL-C, and hs-CRP, were computed using Poisson regression for each of the 3 adjustment approaches. Significance was evaluated for individual quintile groups and for linear trends across quintiles. We also related Lp(a) continuously and dichotomized at 150 nmol/L, and across quintiles, to all clinical and subclinical endpoints using linear, logistic, or Cox regression, as appropriate. The primary outcome was the association between Lp(a) levels at Y7 and incident ASCVD events. Additional analyses examining other cardiovascular and subclinical outcomes were exploratory in nature and therefore not adjusted for multiple comparisons, with nominal *P* values reported to facilitate hypothesis generation for future studies.

For proteomics analyses, we randomly divided the subset of the cohort with proteomic measurements into 70% for the derivation set and 30% for the validation set. We performed linear regression using natural log-transformed and standardized Lp(a) as the predictor and each proteomic feature as the outcome variable (also standardized). These were fitted as unadjusted, age-/sex-/race-adjusted, and fully adjusted models to derive standardized β coefficients. *P* values were adjusted using the Benjamini-Hochberg FDR control method within each predictor set and adjustment approach. These relationships were used to identify possible mechanistic relationships between Lp(a) and candidate effectors.

We also developed signatures of Lp(a) (continuous variable) using LASSO penalized regression with proteomic features as predictors. The LASSO hyperparameter (λ) was optimized through repeated 5-fold cross-validation. The final model retained 62 proteins of the 184 initially measured. The signatures were fitted in the (70%) derivation subset and then applied to both the derivation and validation subsets. Proteomic signatures were subsequently related to outcomes using linear, logistic, and Cox regression analyses, as described above. Models including omics scores and additionally adjusted for Lp(a) were also fitted to compare the scores with measured Lp(a). Statistical analyses were conducted in R, version 4.3.2 (R Foundation for Statistical Computing), with a significance level of 0.05 (type 1 error) and type 1 error handling where noted. Proteins with nominally significant (*P <* 0.05) unadjusted regression coefficients were selected for pathway enrichment analysis using the Reactome database, with the full set of 184 measured Olink proteins serving as the background reference. We elected to use a nominal significance threshold for pathway input rather than FDR-corrected associations because the purpose of this analysis was hypothesis generation rather than formal inference; an FDR threshold would have yielded too few proteins for meaningful enrichment testing. These data were plotted as dot plots, combining Benjamini-Hochberg–adjusted *P* values with the proportion of significant genes in each pathway.

We implemented a revised covariate adjustment structure with 3 tiers: a base age/sex/race model; a partially adjusted model adding Y7 BMI; and a fully adjusted model further including LDL-C, total cholesterol, HDL-C, triglycerides, diabetes status, systolic and diastolic BP, BP medication use, and smoking status. All primary analyses were repeated across all adjustment tiers. As a sensitivity analysis for potential renal confounding, we additionally adjusted fully adjusted models for the eGFR calculated from Y10 serum creatinine using the CKD-EPI 2009 equation (Y7 creatinine was not collected in CARDIA).

Given the high prevalence of zero CAC values (88.9% at Y15), we used 2-part (hurdle) models as our primary analytic approach for CAC outcomes. The first part modeled the probability of any detectable CAC using logistic regression; the second part modeled ln(CAC) among participants with a CAC of greater than 0 using linear regression (Supplemental Table 3).

We evaluated whether adding Lp(a) to each adjustment tier materially improved the prediction of CAC using 3 complementary metrics: C-statistics with DeLong testing, likelihood ratio tests, and information criteria (AIC/BIC) (Supplemental Table 5).

The proportional hazards assumption was evaluated using Schoenfeld residuals (cox.zph) for all Cox models. Given the 7.3% non-CVD mortality rate during follow-up, we also performed Fine-Gray subdistribution hazard regression, treating non-CVD deaths as competing events (Supplemental Tables 4 and 9).

### Study approval.

The CARDIA study was approved by the IRBs at each participating field center (University of Alabama at Birmingham, Northwestern University, University of Minnesota, and Kaiser Permanente). All participants provided written informed consent at each examination.

### Data availability.

Data from the CARDIA study have been deposited in dbGaP (accession nos. phs003440.v1.p1 and phs003439.v1.p1) and are awaiting parent study release. Access can also be obtained from the CARDIA Coordinating Center (https://www.cardia.dopm.uab.edu/) and requires an approved manuscript proposal and data use agreement. UK Biobank data were accessed under application number 7089. UK Biobank is an open-access resource. Further information and the application process are available at https://www.ukbiobank.ac.uk/ Supporting data values for all figures and tables, with separate tabs for each figure panel and supplemental table, are provided in the Supporting Data Values file. This article does not report the original code. The analyses here utilize standard packages in R. Analytics code is available from the corresponding author upon reasonable request.

## Author contributions

SNG and VLM conceived and designed the study, performed the statistical analyses, interpreted the results, and drafted the manuscript. RVS and RSR contributed to study conception and design. SY and LZ contributed to data analysis. TJ contributed to data curation. CJP contributed to the analytic methodology. MKG performed the external replication analysis in the UK Biobank. CC, KT, JEF, and RVS contributed to the generation of the proteomic data, and BT contributed to the laboratory measurement of Lp(a). SNG, VLM, DLJ, DD, DRJ, EJB, SS, MAS, BTE, JTW, RD, and VB contributed to data acquisition. SNG, VLM, RVS, RSR, MKG, CJP, DLJ, DRJ, EJB, SS, JTW, and VB contributed to the interpretation of the data and to the critical revision of the manuscript for important intellectual content. All authors reviewed and approved the final version of the manuscript.

## Conflict of interest

VLM owns stock or stock options in Abbott Laboratories, Abbvie, Advanced Micro Devices, Amgen, Baxter International, Boston Scientific, Bristol-Myers Squibb, Cardinal Health, Cigna, Eli Lilly, GE Healthcare, Intel, Ionetix, Johnson and Johnson, Medtronic, Merck, nVidia, Oracle, Pfizer, Viatris, and Zimmer Biomet. He has received research grants and consulting fees from Siemens Medical Imaging, Jubilant Draximage, and Credence Management Solutions and has also served on medical advisory boards for Ionetix. RVS has served as a consultant for Amgen and Cytokinetics. RVS is supported by grants from the National Institutes of Health. RVS has equity ownership in and is a consultant for Thryv Therapeutics, with support for travel, and is supported by Kardigan. RVS is a co-inventor on pending or issued patents on molecular biomarkers of fitness (19/443,666), dementia, lung disease (19/170,650), cardiovascular diseases and phenotypes (63/755,879), and metabolic health (e.g., liver fat, 19/443,584), and use of RNAs (including spatial RNAs in 63/910,286; 19/571,412) as therapeutics and/or diagnostic biomarkers in disease. EJB has received consulting fees from New Amsterdam Pharmaceuticals. MAS has served as a consultant for Genetic Direction and serves on the scientific advisory board for Elysium Health. RD reports being a scientific co-founder, consultant, and equity holder (pending) for Pensieve Health and a consultant for Variant Bio. VB reports research funding to her institution from the NHLBI through a subcontract from Wake Forest University, Amgen, and Novartis. She serves on data and safety monitoring boards (DSMBs) for the NIA, Eli Lilly, and Verve Therapeutics and has received honoraria from the American College of Cardiology, the American Heart Association, the American Society of Preventive Cardiology, and New Amsterdam Pharma. RSR reports research funding to his institution from Amgen, Arrowhead, Eli Lilly, Merck, NIH, Novartis, Novo Nordisk, Regeneron, and 89Bio. RSR has received consulting fees from Amgen, Avilar, CRISPER Therapeutics, Eli Lilly, Lipigon, New Amsterdam, Novartis, Precision Biosciences, Regeneron, UltraGenyx, and Verve Therapeutics, nonpromotional honoraria from Meda Pharma, and royalties from Wolters Kluwer (UpToDate), and holds stock in MediMergent. He reports patent applications for “Methods and systems for biocellular marker detection and diagnosis using a microfluidic profiling device” (PCT/US2019/026364); “Compositions and methods relating to the identification and treatment of immunothrombotic conditions” (PCT/US2021/63104926); and “Quantification of Lp(a) versus non-Lp(a) ApoB concentration: development of a novel validated equation” (PCT/US2021/63248837).

## Funding support

This work was supported in part by the NIH and is subject to the NIH Public Access Policy. This policy requires that peer-reviewed research papers resulting from NIH-funded research be submitted to PubMed Central upon acceptance for publication. The content is solely the responsibility of the authors and does not necessarily represent the official views of the NIH.

- VA MERIT grant 1I01CX002560 and the Taubman Medical Research Institute (Wolfe Scholarship) (to SNG).
- NIDDK, NIH (U01DK123013-03, to VLM and RVS).
- NIA, NIH (R01AG059729, to VLM and RVS).
- NHLBI, NIH (R01HL136685, to VLM and RVS).
- American Heart Association Strategically Focused Research Network grant in Cardiometabolic Disease (funded proteomics in CARDIA, to VLM and RVS).
- NIH (K23MD017253, to EJB).
- Blue Cross Blue Shield of Michigan Foundation (to EJB).
- NIH (R01HL167733, to BTE).
- A. Alfred Taubman Medical Research Institute (to BTE).
- National Institute of Nursing Research (R01NR019628, to MAS).
- NIGMS, NIH (R35-GM124836, to RD).
- The CARDIA study was conducted and supported by the NHLBI in collaboration with the University of Alabama at Birmingham (75N92023D00002 and 75N92023D00005), Northwestern University (75N92023D00004), University of Minnesota (75N92023D00006), and the Kaiser Foundation Research Institute (75N92023D00003). This manuscript has been reviewed by CARDIA for scientific content.

## Supplementary Material

Supplemental data

ICMJE disclosure forms

Supporting data values

## Figures and Tables

**Table 1:**
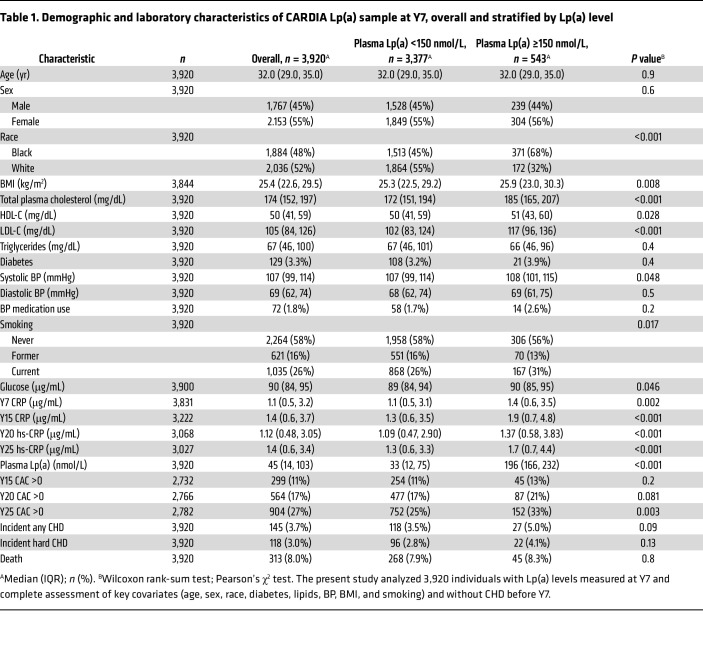
Demographic and laboratory characteristics of CARDIA Lp(a) sample at Y7, overall and stratified by Lp(a) level

## Version 1 04/21/2026

In-Press Preview

## Version 2 06/15/2026

Electronic publication
