# Supplementary material for: Lipoprotein(a)-associated proteomic signature predicts cardiovascular disease in young adults
Source: J Clin Invest. 2026 Apr 16;136(12):e204287. doi: 10.1172/JCI204287 (PMC13262712; doi:10.1172/JCI204287)
Supplement: Supplemental data [file jci-136-204287-s156.pdf]

Contents

1. Supplemental materials and methods.
2. Supplemental Table 1. CARDIA -omics subcohort demographics and laboratory studies.
3. Supplemental Table 2. UK Biobank subcohort demographics and laboratory studies.
4. Supplemental Figure 1. Lp(a) and correlations with lipid and inflammatory parameters.
5. Supplemental Figure 2. Lp(a) and ASCVD endpoints; full CARDIA cohort.
6. Supplemental Figure 3. Lp(a) vs LDL-C vs hs-CRP and ASCVD outcomes.
7. Supplemental Figure 4. Correlation between Lp(a)-associated proteomic scores and Y7 plasma Lp(a).
8. Supplemental Figure 5. Visualization of Lp(a) transformations.
9. Supplemental Table 3. Two-part hurdle models for zero-inflated CAC outcomes.
10. Supplemental Table 4. Cox proportional hazards and competing risk models.
11. Supplemental Table 5. Model comparison statistics.
12. Supplemental Table 6. hsCRP tertile-stratified analyses.
13. Supplemental Table 7. Race-stratified analyses.
14. Supplemental Table 8. eGFR sensitivity analysis.
15. Supplemental Table 9. Event type distribution.

Supplemental materials and methods.

Data Sources and Participants

The UK Biobank represents a prospective cohort study encompassing 502,421 individuals from the general UK population. Between March 2006 and October 2010, participants aged 37–73 years attended one of 22 assessment centers across Scotland, England, and Wales. Each participant completed a touchscreen questionnaire, had physical measurements taken, and provided blood, urine, and saliva samples at baseline. Detailed information about the UKB protocol can be found at http://www.ukbiobank.ac.uk. Participants without CVD at baseline, with proteomics data, and who had measured Lp(a) levels were included. Subjects who had proteomics data with > 20 % of the 61 proteins (of 62 identified by LASSO in CARDIA, with MMP-2 unavailable on the Olink Explore 1536 platform) from the Lp(a)-associated proteomic score were excluded, resulting in a final study population of 37,996. Four outcomes were assessed over a 13.6-year median follow-up: incident hard CHD (ICD-10: I20.0, I21, I22, I23; CHD deaths: I20-I22, I24-I25), incident any CHD (hard CHD plus revascularization, OPCS-4: K40-K46, K49-K50), all-cause death, and baseline CRP levels. Cox proportional hazards models were used for time-to-event outcomes, and linear regression for CRP. Adjustments were applied at three levels: 1) unadjusted, 2) adjusted for age, sex, and ethnicity, 3) fully adjusted (adding total cholesterol, HDL-C, LDL-C, triglycerides, diabetes, systolic/diastolic blood pressure, antihypertensive medication, and smoking status); all continuous variables were scaled to 0 mean and 1 SD.

Plasma Proteomics in UK Biobank

Blood samples were primarily collected from UKB participants during their baseline visit. Plasma proteome profiling was executed utilizing the antibody-based Olink® Proteomics PEA technology. Protein levels were provided as Normalized Protein eXpression (NPX) values, generated by log-transforming counts normalized to external controls. Assessments indicated that protein expression levels were minimally affected by protein batch, study center, and genetic principal components. Detailed protocols for sample handling, processing, and quality control are available online. The Lp(a)-associated proteomic score was calculated using 61 of the 62 CARDIA-derived proteins available on the

UK Biobank Olink Explore 1536 platform (MMP-2 unavailable). NPX values of proteins were standardized to a mean of 0 and 1 SD prior to score calculation. The final study population was divided 70 % / 30 %, stratified by quartiles of ln-Lp(a) to keep the same distribution of the target variable in both subsets. Within the 70 % derivation subset, a LASSO model ($\alpha = 1$) was fitted to predict Z-scaled ln-Lp(a); λ was selected by an internal 5-fold cross-validation. The original CARDIA-derived LASSO coefficients were reweighted in the UK Biobank derivation subset to account for platform differences; these reweighted β values were applied to all participants. Scores were Z-scaled separately within the derivation and validation subsets before any downstream analysis. The reweighted score yielded a Pearson correlation of $r = 0.37$ with ln-transformed Lp(a) in the full sample ($R^2 = 0.137$ for rank-normalized Lp(a); $R^2 = 0.102$ for continuous Lp(a)).

Supplemental Table 1. Demographic and laboratory characteristics of CARDIA Lp(a) multi-omics sample at Year 7, overall and stratified by Lp(a) level. The omics study analyzes 2,303 individuals with Lp(a) levels measured at Year 7 and complete assessment of key covariates (age, sex, race, diabetes, lipids, blood pressure, BMI, and smoking), metabolomics and proteomics measures and without CHD before Year 7.

Characteristic	N	Overall	derivation	validation	p-value[2]
		N = 2,303[1]	N = 1,612[1]	N = 691[1]	
Y7 Age (y)	2,303	33.0 (29.0, 35.0)	32.0 (29.0, 35.0)	33.0 (30.0, 35.0)	0.062
SEX	2,303				>0.9
male		1,263 (55%)	883 (55%)	380 (55%)	
female		1,040 (45%)	729 (45%)	311 (45%)	
RACE	2,303				0.4
black		1,028 (45%)	728 (45%)	300 (43%)	
white		1,275 (55%)	884 (55%)	391 (57%)	
Y7 BMI (kg/m2)	2,280	24.8 (22.4, 27.9)	24.9 (22.5, 28.0)	24.6 (22.1, 27.7)	0.061
Y7 Total Plasma Cholesterol (m	2,303	173 (154, 195)	173 (154, 195)	174 (154, 198)	0.5
Y7 Total HDL Cholesterol (mg/dl	2,303	52 (44, 62)	52 (44, 62)	53 (44, 63)	0.3
Y7 LDL Cholesterol (mg/dl)	2,303	103 (85, 126)	103 (84, 126)	103 (85, 126)	0.7
Y7 Plasma LDL Quintile	2,303				0.7
Q1		480 (21%)	343 (21%)	137 (20%)	
Q2		510 (22%)	346 (21%)	164 (24%)	
Q3		441 (19%)	315 (20%)	126 (18%)	
Q4		448 (19%)	316 (20%)	132 (19%)	
Q5		424 (18%)	292 (18%)	132 (19%)	
Y7 Triglycerides (mg/dl)	2,303	63 (45, 91)	64 (45, 91)	63 (45, 92)	0.6
Y7 Diabetes	2,303	48 (2.1%)	40 (2.5%)	8 (1.2%)	0.042
Y7 Avg SBP (mmHg)	2,303	107 (101, 115)	107 (101, 115)	107 (100, 114)	0.4
Y7 Avg DBP (mmHg)	2,303	68 (63, 74)	68 (63, 74)	68 (62, 74)	0.7
Y7 BP Medication Use	2,303	24 (1.0%)	19 (1.2%)	5 (0.7%)	0.3
Y7 Smoking	2,303				0.7
never		1,404 (61%)	986 (61%)	418 (60%)	
former		367 (16%)	250 (16%)	117 (17%)	
current		532 (23%)	376 (23%)	156 (23%)	
Y7 NIST Standard Recalibrated	2,296	90 (85, 95)	90 (85, 95)	89 (85, 95)	0.5
Y7 CRP (BNII Method) (ug/ml)	2,294	0.90 (0.42, 2.40)	0.90 (0.42, 2.41)	0.89 (0.40, 2.38)	0.7
Y7 Plasma CRP Quintile	2,294				0.7
Q1		531 (23%)	363 (23%)	168 (24%)	
Q2		513 (22%)	369 (23%)	144 (21%)	
Q3		489 (21%)	342 (21%)	147 (21%)	
Q4		399 (17%)	275 (17%)	124 (18%)	
Q5		362 (16%)	258 (16%)	104 (15%)	
Y15 CRP (BNII Method) (ug/dl)	1,983	1.2 (0.5, 3.2)	1.2 (0.5, 3.2)	1.1 (0.5, 3.0)	0.3
Y20 hsCRP (ug/ml)	1,904	1.01 (0.45, 2.52)	1.00 (0.45, 2.51)	1.09 (0.46, 2.55)	0.6
Y25 hsCRP (ug/ml)	1,869	1.2 (0.6, 2.9)	1.3 (0.6, 3.0)	1.2 (0.6, 2.8)	0.8
Y7 Plasma Lp(a) (nmol/l)	2,303	39 (13, 99)	39 (13, 99)	40 (13, 96)	>0.9
Y7 Plasma Lp(a) Quintile	2,303				0.6
Q1		510 (22%)	362 (22%)	148 (21%)	
Q2		485 (21%)	337 (21%)	148 (21%)	
Q3		440 (19%)	309 (19%)	131 (19%)	
Q4		439 (19%)	295 (18%)	144 (21%)	
Q5		429 (19%)	309 (19%)	120 (17%)	
Y7 Plasma Lp(a) ≥ 150 nmol/l	2,303	300 (13%)	219 (14%)	81 (12%)	0.2
Y15 ln(CAC+1)	1,699	0.00 (0.00, 0.00)	0.00 (0.00, 0.00)	0.00 (0.00, 0.00)	0.2
Y20 ln(CAC+1)	1,720	0.00 (0.00, 0.00)	0.00 (0.00, 0.00)	0.00 (0.00, 0.00)	>0.9
Y25 ln(CAC+1)	1,706	0.00 (0.00, 1.65)	0.00 (0.00, 1.59)	0.00 (0.00, 1.83)	>0.9
Y7 ln(C-Reactive Protein)	2,294	-0.11 (-0.87, 0.88)	-0.11 (-0.87, 0.88)	-0.12 (-0.92, 0.87)	0.7
Y15 ln(C-Reactive Protein)	1,983	0.18 (-0.65, 1.15)	0.22 (-0.63, 1.17)	0.13 (-0.73, 1.09)	0.3
Y20 ln(C-Reactive Protein)	1,904	0.01 (-0.80, 0.92)	-0.01 (-0.81, 0.92)	0.08 (-0.78, 0.93)	0.6
Y25 ln(C-Reactive Protein)	1,869	0.22 (-0.56, 1.06)	0.25 (-0.58, 1.09)	0.18 (-0.53, 1.04)	0.8
Incident Any CHD	2,303	71 (3.1%)	49 (3.0%)	22 (3.2%)	0.9
Incident Hard CHD	2,303	53 (2.3%)	37 (2.3%)	16 (2.3%)	>0.9
Death	2,303	141 (6.1%)	96 (6.0%)	45 (6.5%)	0.6
Incident Any CHD Follow-up Ye	2,303	27.15 (26.81, 28.91)	27.16 (26.81, 28.91)	27.10 (26.81, 28.94	>0.9
Incident Hard CHD Follow-up Y	2,303	27.16 (26.82, 28.92)	27.18 (26.81, 28.91)	27.12 (26.82, 28.9	>0.9
Death Follow-up Years	2,303	27.19 (26.83, 28.93)	27.20 (26.83, 28.92)	27.15 (26.84, 28.97	>0.9

[1] Median (Q1, Q3); n (%)
[2] Wilcoxon rank sum test; Pearson's Chi-squared test

Supplemental Table 2. Demographic and laboratory characteristics of UK Biobank subcohort.

Characteristic	N = 37,996
Age at recruitment (years), Median (Q1, Q3)	58.00 (50.00, 63.00)
Sex, n (%)	
Female	20,822 (55%)
Male	17,174 (45%)
Insulin use, n (%)	1,348 (3.5%)
Diabetes, n (%)	1,823 (4.8%)
Hypertension, n (%)	10,059 (26%)
BMI (kg/m^2), Median (Q1, Q3)	26.66 (24.10, 29.76)
Diastolic blood pressure (mmHg), Median (Q1, Q3)	82.00 (75.00, 89.00)
Systolic blood pressure (mmHg), Median (Q1, Q3)	136.00 (124.50, 149.00)
C-reactive protein (mg/L), Median (Q1, Q3)	1.33 (0.66, 2.78)
Lipoprotein A (nmol/L), Median (Q1, Q3)	21.50 (9.60, 63.00)
Cholesterol (mg), Median (Q1, Q3)	5.64 (4.91, 6.41)
HDL Cholesterol (mmol/L), Median (Q1, Q3)	1.40 (1.17, 1.68)
LDL Cholesterol (mmol/L), Median (Q1, Q3)	3.51 (2.95, 4.12)
Triglycerides (mmol/L), Median (Q1, Q3)	1.47 (1.04, 2.13)
Glycated haemoglobin (mmol/mol), Median (Q1, Q3)	35.30 (32.80, 37.90)
Current smoking, n (%)	4,007 (11%)
Blood Pressure Medication, n (%)	8,570 (23%)
Cholesterol-lowering Medication, n (%)	6,324 (17%)

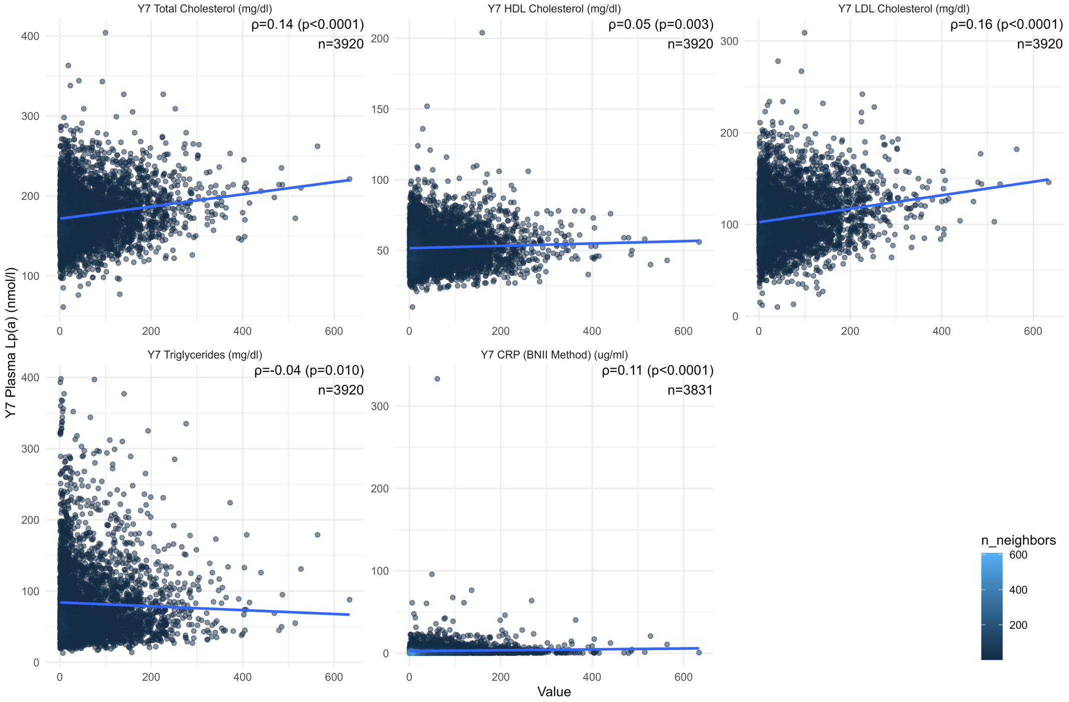

Supplemental Figure 1. Scatter plots relating plasma Lp(a) (vertical axis) to total cholesterol, HDL cholesterol, LDL cholesterol, triglycerides, and high-sensitivity C-reactive protein with linear best-fit lines shown.

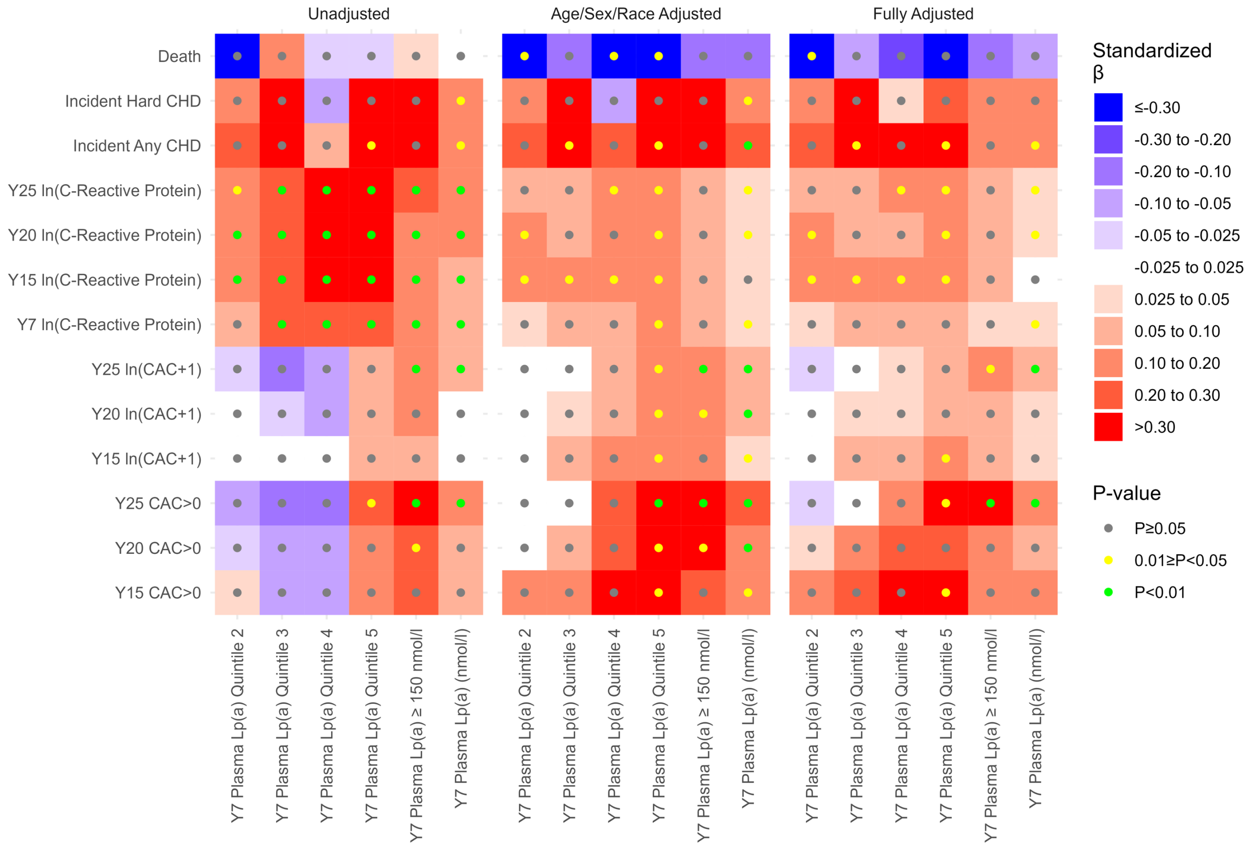

Supplemental Figure 2. Relationships between Lp(a) levels and coronary calcification, inflammation and outcomes. Heatmap of standardized regression coefficients from models relating Y7 Lp(a) variables (quintiles, 150 nmol/L threshold, and as a continuous variable) to CAC, hs-CRP, and CHD endpoints using unadjusted linear, logistic and Cox regression. Values for regression coefficient directionality (color), magnitude (color gradient), and statistical significance (color of circle) are depicted in the figure legend. This figure is similar to Figure 1 from the manuscript except these data present the unadjusted and adjusted models.

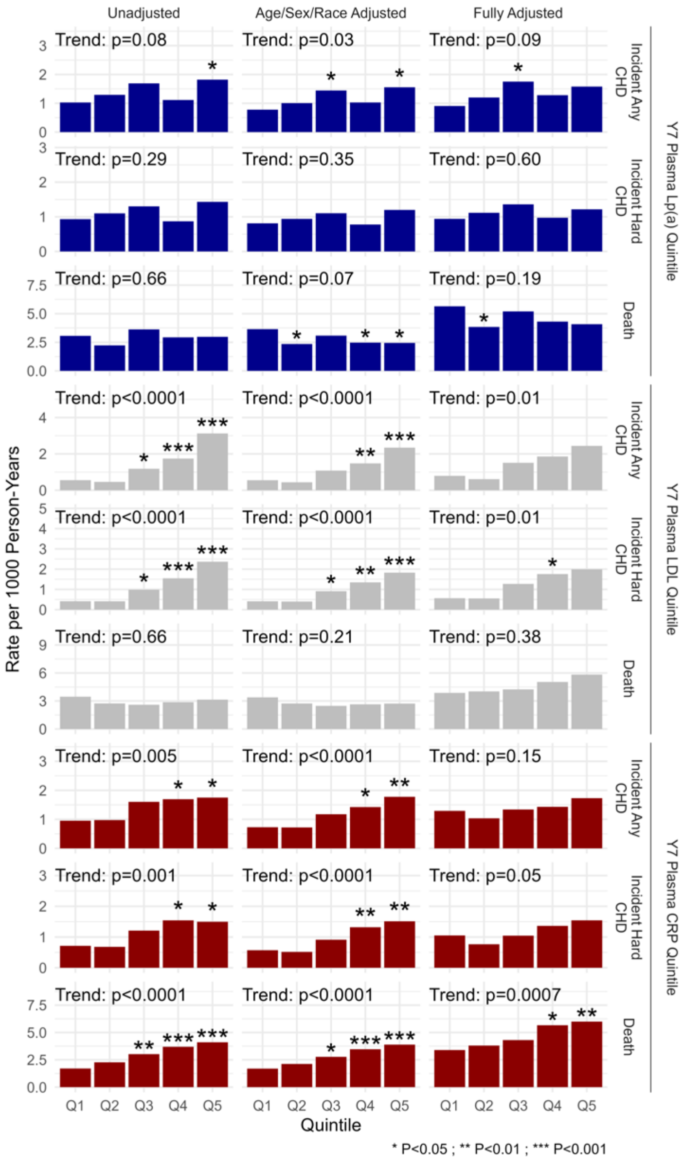

Supplemental Figure 3. Event rates per 1000 person-year for incident any CHD, incident hard CHD, and death from any cause across quintiles of Lp(a) (blue), LDL cholesterol (grey) , and high-sensitivity CRP (red). Statistical significance was computed using Poisson regression and is expressed for a linear trend across quintiles in text labels and for individual quintiles using * for P<0.05, ** for P<0.01, and *** for P<0.001.

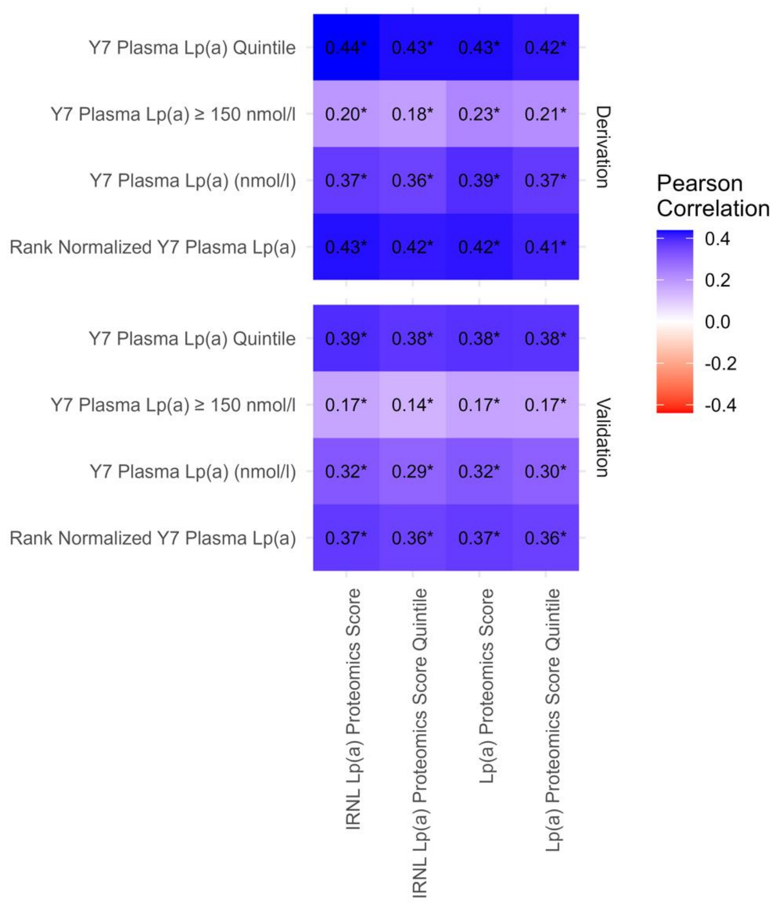

Supplemental Figure 4. Correlations between Lp(a) proteomic score (continuously and as quintiles) with measured Lp(a) (continuously, dichotomized, and as quintiles) in both the omics derivation and validation subcohorts.

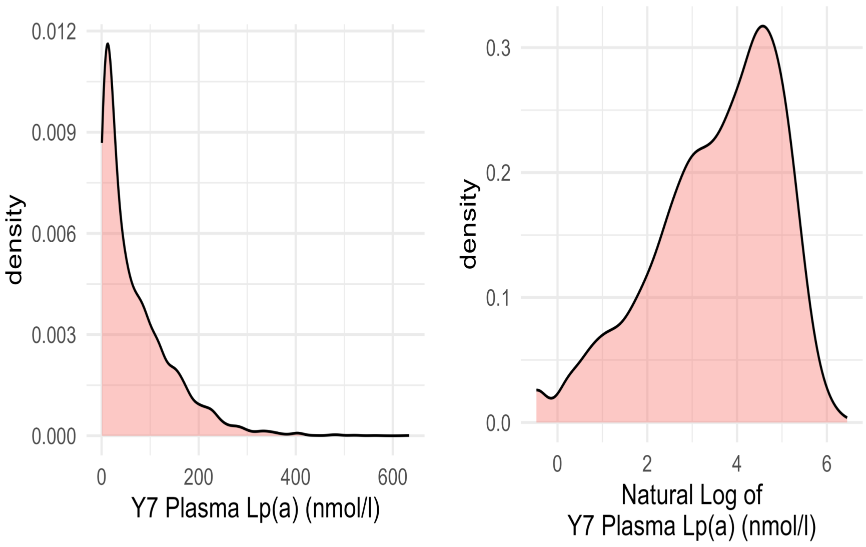

Supplemental Figure 5. Lp(a) exhibited a significantly right-skewed distribution (left panel). As such our primary analysis used the natural log transformation to improve normality (right panel). As a sensitivity analysis, inverse rank normalized Lp(a) was also

Supplemental Table 3. Two-part hurdle models for zero-inflated CAC outcomes. Among the 3,043 participants with CAC data available, 2,706 (88.9%) had CAC = 0 and 337 (11.1%) had CAC > 0. Part 1 models the probability of any detectable CAC (CAC > 0) using logistic regression; Part 2 models ln(CAC) among participants with CAC > 0 using linear regression. Results are presented across four adjustment tiers.

Adjustment	Presence OR	Presence 95% CI Lower	Presence 95% CI Upper	Presence P	Presence N	Severity Beta	Severity 95% CI Lower	Severity 95% CI Upper	Severity P	Severity N
**Unadjusted**	1.0008	0.9991	1.0024	0.3406	2690.0	0.0006	-0.0017	0.0028	0.6351	298.0
**Age/Sex/Race**	1.0019	1.0002	1.0036	0.0281	2690.0	0.0003	-0.0021	0.0026	0.8302	298.0
**Partially Adjusted**	1.0020	1.0002	1.0037	0.0225	2690.0	0.0001	-0.0023	0.0024	0.9494	298.0
**Fully Adjusted**	1.0016	0.9997	1.0033	0.0869	2690.0	-0.0002	-0.0026	0.0021	0.8490	298.0

Supplemental Table 4. Cox proportional hazards, proportional hazards assumption testing, and Fine-Gray competing risk models. Outcome is adjudicated CHD events (myocardial infarction, coronary revascularization, or CHD death) through Year 35 follow-up.

Adjustment	Cox HR	Cox 95% CI Lower	Cox 95% CI Upper	Cox P	Cox N	Cox Events	PH Lp(a) P	PH Global P	Fine-Gray SHR	FG 95% CI Lower	FG 95% CI Upper	FG P	FG N	FG CVD Events	FG Non-CVD Deaths
**Unadjusted**	1.0017	1.0003	1.0031	0.0147	3958.0	297.0	0.7629	0.7629	1.0017	1.0004	1.0030	0.0093	3958.0	297.0	226.0
**Age/Sex/Race**	1.0009	0.9995	1.0024	0.2140	3958.0	297.0	0.7711	0.0880	1.0011	0.9996	1.0025	0.1480	3958.0	297.0	226.0
**Partially Adjusted**	1.0011	0.9996	1.0025	0.1530	3878.0	296.0	0.7688	0.0825	1.0012	0.9997	1.0026	0.1080	3878.0	296.0	222.0
**Fully Adjusted**	1.0006	0.9991	1.0021	0.4090	3846.0	287.0	0.6724	0.0043	1.0007	0.9993	1.0022	0.3190	3846.0	287.0	217.0

Supplemental Table 5. Model comparison statistics. C-statistics with 95% confidence intervals, likelihood ratio test statistics, and information criteria (AIC, BIC) for nested Cox proportional hazards models of incident CHD.

Adjustment	Base C-statistic	Enhanced C-statistic	Delta C	DeLong P	N	LRT Chi-sq	LRT df	LRT P	Delta AIC	Delta BIC
**Unadjusted**	0.5000	0.5087	0.0087	0.6240	2754.0	0.7000	1.0000	0.4030	1.3000	7.2000
**Age/Sex/Race**	0.7159	0.7181	0.0022	0.3318	2754.0	4.2200	1.0000	0.0399	-2.2000	3.7000
**Partially Adjusted**	0.7270	0.7292	0.0022	0.3264	2711.0	4.9100	1.0000	0.0268	-2.9000	3.0000
**Fully Adjusted**	0.7687	0.7696	0.0009	0.5269	2690.0	2.8400	1.0000	0.0919	-0.8000	5.1000

Supplemental Table 6. hsCRP tertile-stratified analyses with interaction testing. Participants were stratified by high-sensitivity C-reactive protein (hsCRP) tertiles measured at Year 7.

hsCRP Tertile	Tertile Median (ug/mL)	Tertile Range	CAC Presence N	CAC Presence OR	CAC Presence 95% CI Lower	CAC Presence 95% CI Upper	CAC Presence P	CAC Severity N	CAC Severity Beta	CAC Severity 95% CI Lower	CAC Severity 95% CI Upper	CAC Severity P
**Low**	0.3400	0.15-0.64	920.0	1.0003	0.9970	1.0034	0.8300	96.0000	-0.0006	-0.0051	0.0039	0.7890
**Intermediate**	1.1300	0.64-2.21	895.0	1.0032	1.0002	1.0060	0.0297	105.0	-0.0001	-0.0038	0.0036	0.9550
**High**	4.6100	2.21-333.0	837.0	1.0025	0.9994	1.0055	0.1090	99.0000	0.0019	-0.0025	0.0062	0.4050
**Interaction Tests**												
**Endpoint**	Interaction P	Significant										
**CAC Presence**	0.8849	No										
**CAC Severity**	0.3937	No										

Supplemental Table 7. Race-stratified analyses with interaction testing. CARDIA enrolled approximately equal numbers of Black and White participants by design. Cox proportional hazards models were fit separately within each racial group.

Endpoint	Race	N	Events/N with CAC>0	Estimate	Measure	95% CI Lower	95% CI Upper	P
**CAC Presence**	White	1527.0	212.0	1.0018	OR	0.9994	1.0042	0.1397
**CAC Presence**	Black	1184.0	125.0	1.0022	OR	0.9997	1.0047	0.0807
**CAC Severity (CAC>0)**	White	200.0	212.0	0.0008	Beta	-0.0024	0.0040	0.6136
**CAC Severity (CAC>0)**	Black	102.0	125.0	0.0001	Beta	-0.0036	0.0037	0.9790
**CVD Events**	White	2018.0	122.0	1.0027	HR	1.0003	1.0052	0.0304
**CVD Events**	Black	1860.0	174.0	1.0003	HR	0.9984	1.0021	0.7760
**hsCRP**	White	1982.0		-0.0002	Beta	-0.0010	0.0006	0.6184
**hsCRP**	Black	1807.0		0.0010	Beta	0.0004	0.0017	0.0023
**Formal Interaction Tests (DL6LPA x RACE)**								
**Endpoint**	Interaction Term	Interaction P	Significant					
**CAC Presence**	DL6LPA:RACEwhite	0.7299	No					
**CAC Severity (CAC>0)**	DL6LPA:RACEwhite	0.7824	No					
**CVD Events**	DL6LPA:RACEwhite	0.1237	No					
**hsCRP**	DL6LPA:RACEwhite	0.0206	Yes					

Supplemental Table 8. eGFR sensitivity analysis. Sensitivity analysis incorporating Year 10 creatinine-based estimated glomerular filtration rate (eGFR, CKD-EPI equation) as an additional covariate.

Endpoint	Measure	Fully Adjusted Effect (95% CI)	Fully Adjusted P	Fully Adjusted N	Fully Adjusted + eGFR Effect (95% CI)	Fully Adjusted + eGFR P	Fully Adjusted + eGFR N	% Change	Attenuation
**CAC Presence**	OR	1.0014 (0.9995, 1.0033)	0.1464	2529.0	1.0014 (0.9995, 1.0033)	0.1455	2529.0	0.0004	Negligible (<5%)
**CAC Severity (CAC>0)**	Beta	-0.0006 (-0.0029, 0.0018)	0.6320	280.0	-0.0006 (-0.0030, 0.0018)	0.6180	280.0	-4.4506	Negligible (<5%)
**CVD Events**	HR	1.0008 (0.9992, 1.0024)	0.3283	3389.0	1.0008 (0.9992, 1.0023)	0.3237	3389.0	0.0004	Negligible (<5%)
**hsCRP**	Beta	0.0005 (0.0000, 0.0011)	0.0461	3314.0	0.0005 (0.0000, 0.0011)	0.0455	3314.0	0.2941	Negligible (<5%)
**CKD Prevalence Summary**									
**CKD Stage**	Year 0 N	Year 0 %	Year 10 N	Year 10 %					
**Stage 1 (>=90)**	3023.0	59.1%	2510.0	70.3%					
**Stage 2 (60-89)**	1986.0	38.8%	1045.0	29.3%					
**Stage 3a (45-59)**	33.0000	0.6%	12.0000	0.3%					
**Stage 3b (30-44)**	4.0000	0.1%	1.0000	0.0%					
**Stage 4 (15-29)**	0.0000	0.0%	1.0000	0.0%					
**Stage 5 (<15)**	3.0000	0.1%	2.0000	0.0%					
**Total Stage 3+**	40.0000	0.79%	16.0000	0.45%					
**Year 10 eGFR: Mean 98.9 +/- 16.7 mL/min/1.73m2, Median 97.1, N=3,571**									
**eGFR-Lp(a) correlation: Pearson r = 0.06, p = 0.0003**									

Supplemental Table 9. Event type distribution. Distribution of event types in the full Lp(a) analytic cohort (N=2,299) and within the CAC subcohort (N=3,043).

Event Type	N	%
**Censored (no event)**	4356.0	85.2%
**CVD event (primary outcome)**	387.0	7.6%
**Non-CVD death (competing risk)**	371.0	7.3%
**Total**	5114.0	100.0%
