# Supplementary material for: Lipoprotein(a)-associated proteomic signature predicts cardiovascular disease in young adults
Source: J Clin Invest. 2026 Apr 16;136(12):e204287. doi: 10.1172/JCI204287 (PMC13262712; doi:10.1172/JCI204287)
Supplement: ICMJE disclosure forms [file jci-136-204287-s157.pdf]

ICMJE DISCLOSURE FORM

Date:<br>March 8, 2026	Click or tap to enter a date.
Your Name:<br>Sascha N. Goonewardena	Click or tap here to enter text.
Manuscript Title:<br>Plasma Proteomic Signatures of Lipoprotein(a) and Cardiovascular Disease in Young Adults	Click or tap here to enter text.
Manuscript Number (if known):<br>204287-JCI-CRPH-1	Click or tap here to enter text.

In the interest of transparency, we ask you to disclose all relationships/activities/interests listed below that are related to the content of your manuscript. "Related" means any relation with for-profit or not-for-profit third parties whose interests may be affected by the content of the manuscript. Disclosure represents a commitment to transparency and does not necessarily indicate a bias. If you are in doubt about whether to list a relationship/activity/interest, it is preferable that you do so.

The author's relationships/activities/interests should be defined broadly. For example, if your manuscript pertains to the epidemiology of hypertension, you should declare all relationships with manufacturers of antihypertensive medication, even if that medication is not mentioned in the manuscript.

In item #1 below, report all support for the work reported in this manuscript without time limit. For all other items, the time frame for disclosure is the past 36 months.

		Name all entities with whom you have this relationship or indicate none (add rows as needed)	Specifications/Comments (e.g., if payments were made to you or to your institution)
**Time frame: Since the initial planning of the work**			
**1**	All support for the present manuscript (e.g., funding, provision of study materials, medical writing, article processing charges, etc.)<br>**No time limit for this item.**	☐ **None**<br>VA MERIT grant 1I01CX002560, Taubman Medical Research Institute (Wolfe Scholarship)	
			Click the tab key to add additional rows.
**Time frame: past 36 months**			
**2**	Grants or contracts from any entity (if not indicated in	☐ **None**<br>None	

		Name all entities with whom you have this relationship or indicate none (add rows as needed)	Specifications/Comments (e.g., if payments were made to you or to your institution)
	item #1 above).		
**3**	Royalties or licenses	☐ **None**<br>None	
**4**	Consulting fees	☐ **None**<br>None	
**5**	Payment or honoraria for lectures, presentations, speakers bureaus, manuscript writing or educational events	☐ **None**<br>None	
**6**	Payment for expert testimony	☐ **None**<br>None	
**7**	Support for attending meetings and/or travel	☐ **None**<br>None	
**8**	Patents planned, issued or pending	☐ **None**<br>None	
**9**	Participation on a Data Safety Monitoring Board or Advisory Board	☐ **None**<br>None	
**10**	Leadership	☐ **None**	

		Name all entities with whom you have this relationship or indicate none (add rows as needed)	Specifications/Comments (e.g., if payments were made to you or to your institution)
	or fiduciary role in other board, society, committee or advocacy group, paid or unpaid	None	
**11**	Stock or stock options	☐ **None**<br>None	
**12**	Receipt of equipment, materials, drugs, medical writing, gifts or other services	☐ **None**<br>None	
**13**	Other financial or non-financial interests	☐ **None**<br>None	

Please place an "X" next to the following statement to indicate your agreement:

☒ X

ICMJE DISCLOSURE FORM

Date:<br>March 8, 2026	Click or tap to enter a date.
Your Name:<br>Shanshan Yao	Click or tap here to enter text.
Manuscript Title:<br>Plasma Proteomic Signatures of Lipoprotein(a) and Cardiovascular Disease in Young Adults	Click or tap here to enter text.
Manuscript Number (if known):<br>204287-JCI-CRPH-1	Click or tap here to enter text.

In the interest of transparency, we ask you to disclose all relationships/activities/interests listed below that are related to the content of your manuscript. "Related" means any relation with for-profit or not-for-profit third parties whose interests may be affected by the content of the manuscript. Disclosure represents a commitment to transparency and does not necessarily indicate a bias. If you are in doubt about whether to list a relationship/activity/interest, it is preferable that you do so.

The author's relationships/activities/interests should be defined broadly. For example, if your manuscript pertains to the epidemiology of hypertension, you should declare all relationships with manufacturers of antihypertensive medication, even if that medication is not mentioned in the manuscript.

In item #1 below, report all support for the work reported in this manuscript without time limit. For all other items, the time frame for disclosure is the past 36 months.

		Name all entities with whom you have this relationship or indicate none (add rows as needed)	Specifications/Comments (e.g., if payments were made to you or to your institution)
**Time frame: Since the initial planning of the work**			
**1**	All support for the present manuscript (e.g., funding, provision of study materials, medical writing, article processing charges, etc.) **No time limit for this item.**	☐ **None**<br>None	
			Click the tab key to add additional rows.
**Time frame: past 36 months**			
**2**	Grants or contracts from any entity (if not indicated in	☐ **None**<br>None	

		Name all entities with whom you have this relationship or indicate none (add rows as needed)	Specifications/Comments (e.g., if payments were made to you or to your institution)
	item #1 above).		
**3**	Royalties or licenses	☐ **None**<br>None	
**4**	Consulting fees	☐ **None**<br>None	
**5**	Payment or honoraria for lectures, presentations, speakers bureaus, manuscript writing or educational events	☐ **None**<br>None	
**6**	Payment for expert testimony	☐ **None**<br>None	
**7**	Support for attending meetings and/or travel	☐ **None**<br>None	
**8**	Patents planned, issued or pending	☐ **None**<br>None	
**9**	Participation on a Data Safety Monitoring Board or Advisory Board	☐ **None**<br>None	
**10**	Leadership	☐ **None**	

		Name all entities with whom you have this relationship or indicate none (add rows as needed)	Specifications/Comments (e.g., if payments were made to you or to your institution)
	or fiduciary role in other board, society, committee or advocacy group, paid or unpaid	None	
**11**	Stock or stock options	☐ **None**<br>None	
**12**	Receipt of equipment, materials, drugs, medical writing, gifts or other services	☐ **None**<br>None	
**13**	Other financial or non-financial interests	☐ **None**<br>None	

Please place an "X" next to the following statement to indicate your agreement:

☒ X

ICMJE DISCLOSURE FORM

Date:<br>March 8, 2026	Click or tap to enter a date.
Your Name:<br>Lanyue Zhang	Click or tap here to enter text.
Manuscript Title:<br>Plasma Proteomic Signatures of Lipoprotein(a) and Cardiovascular Disease in Young Adults	Click or tap here to enter text.
Manuscript Number (if known):<br>204287-JCI-CRPH-1	Click or tap here to enter text.

In the interest of transparency, we ask you to disclose all relationships/activities/interests listed below that are related to the content of your manuscript. "Related" means any relation with for-profit or not-for-profit third parties whose interests may be affected by the content of the manuscript. Disclosure represents a commitment to transparency and does not necessarily indicate a bias. If you are in doubt about whether to list a relationship/activity/interest, it is preferable that you do so.

The author's relationships/activities/interests should be defined broadly. For example, if your manuscript pertains to the epidemiology of hypertension, you should declare all relationships with manufacturers of antihypertensive medication, even if that medication is not mentioned in the manuscript.

In item #1 below, report all support for the work reported in this manuscript without time limit. For all other items, the time frame for disclosure is the past 36 months.

		Name all entities with whom you have this relationship or indicate none (add rows as needed)	Specifications/Comments (e.g., if payments were made to you or to your institution)
**Time frame: Since the initial planning of the work**			
**1**	All support for the present manuscript (e.g., funding, provision of study materials, medical writing, article processing charges, etc.) **No time limit for this item.**	☐ **None**<br>None	
			Click the tab key to add additional rows.
**Time frame: past 36 months**			
**2**	Grants or contracts from any entity (if not indicated in	☐ **None**<br>None	

		Name all entities with whom you have this relationship or indicate none (add rows as needed)	Specifications/Comments (e.g., if payments were made to you or to your institution)
	item #1 above).		
3	Royalties or licenses	☐ **None**<br>None	
4	Consulting fees	☐ **None**<br>None	
5	Payment or honoraria for lectures, presentations, speakers bureaus, manuscript writing or educational events	☐ **None**<br>None	
6	Payment for expert testimony	☐ **None**<br>None	
7	Support for attending meetings and/or travel	☐ **None**<br>None	
8	Patents planned, issued or pending	☐ **None**<br>None	
9	Participation on a Data Safety Monitoring Board or Advisory Board	☐ **None**<br>None	
10	Leadership	☐ **None**	

		Name all entities with whom you have this relationship or indicate none (add rows as needed)	Specifications/Comments (e.g., if payments were made to you or to your institution)
	or fiduciary role in other board, society, committee or advocacy group, paid or unpaid	None	
**11**	Stock or stock options	☐ **None**<br>None	
**12**	Receipt of equipment, materials, drugs, medical writing, gifts or other services	☐ **None**<br>None	
**13**	Other financial or non-financial interests	☐ **None**<br>None	

Please place an "X" next to the following statement to indicate your agreement:

☒ X

ICMJE DISCLOSURE FORM

Date:<br>March 8, 2026	Click or tap to enter a date.
Your Name:<br>Tomasz Jurga	Click or tap here to enter text.
Manuscript Title:<br>Plasma Proteomic Signatures of Lipoprotein(a) and Cardiovascular Disease in Young Adults	Click or tap here to enter text.
Manuscript Number (if known):<br>204287-JCI-CRPH-1	Click or tap here to enter text.

In the interest of transparency, we ask you to disclose all relationships/activities/interests listed below that are related to the content of your manuscript. "Related" means any relation with for-profit or not-for-profit third parties whose interests may be affected by the content of the manuscript. Disclosure represents a commitment to transparency and does not necessarily indicate a bias. If you are in doubt about whether to list a relationship/activity/interest, it is preferable that you do so.

The author's relationships/activities/interests should be defined broadly. For example, if your manuscript pertains to the epidemiology of hypertension, you should declare all relationships with manufacturers of antihypertensive medication, even if that medication is not mentioned in the manuscript.

In item #1 below, report all support for the work reported in this manuscript without time limit. For all other items, the time frame for disclosure is the past 36 months.

		Name all entities with whom you have this relationship or indicate none (add rows as needed)	Specifications/Comments (e.g., if payments were made to you or to your institution)
**Time frame: Since the initial planning of the work**			
**1**	All support for the present manuscript (e.g., funding, provision of study materials, medical writing, article processing charges, etc.) **No time limit for this item.**	☐ **None**<br>None	
			Click the tab key to add additional rows.
**Time frame: past 36 months**			
**2**	Grants or contracts from any entity (if not indicated in	☐ **None**<br>None	

		Name all entities with whom you have this relationship or indicate none (add rows as needed)	Specifications/Comments (e.g., if payments were made to you or to your institution)
	item #1 above).		
3	Royalties or licenses	☐ **None**<br>None	
4	Consulting fees	☐ **None**<br>None	
5	Payment or honoraria for lectures, presentations, speakers bureaus, manuscript writing or educational events	☐ **None**<br>None	
6	Payment for expert testimony	☐ **None**<br>None	
7	Support for attending meetings and/or travel	☐ **None**<br>None	
8	Patents planned, issued or pending	☐ **None**<br>None	
9	Participation on a Data Safety Monitoring Board or Advisory Board	☐ **None**<br>None	
10	Leadership	☐ **None**	

		Name all entities with whom you have this relationship or indicate none (add rows as needed)	Specifications/Comments (e.g., if payments were made to you or to your institution)
	or fiduciary role in other board, society, committee or advocacy group, paid or unpaid	None	
**11**	Stock or stock options	☐ **None**<br>None	
**12**	Receipt of equipment, materials, drugs, medical writing, gifts or other services	☐ **None**<br>None	
**13**	Other financial or non-financial interests	☐ **None**<br>None	

Please place an "X" next to the following statement to indicate your agreement:

☒ X

ICMJE DISCLOSURE FORM

Date:<br>March 8, 2026	Click or tap to enter a date.
Your Name:<br>Donald Lloyd-Jones	Click or tap here to enter text.
Manuscript Title:<br>Plasma Proteomic Signatures of Lipoprotein(a) and Cardiovascular Disease in Young Adults	Click or tap here to enter text.
Manuscript Number (if known):<br>204287-JCI-CRPH-1	Click or tap here to enter text.

In the interest of transparency, we ask you to disclose all relationships/activities/interests listed below that are related to the content of your manuscript. "Related" means any relation with for-profit or not-for-profit third parties whose interests may be affected by the content of the manuscript. Disclosure represents a commitment to transparency and does not necessarily indicate a bias. If you are in doubt about whether to list a relationship/activity/interest, it is preferable that you do so.

The author's relationships/activities/interests should be defined broadly. For example, if your manuscript pertains to the epidemiology of hypertension, you should declare all relationships with manufacturers of antihypertensive medication, even if that medication is not mentioned in the manuscript.

In item #1 below, report all support for the work reported in this manuscript without time limit. For all other items, the time frame for disclosure is the past 36 months.

		Name all entities with whom you have this relationship or indicate none (add rows as needed)	Specifications/Comments (e.g., if payments were made to you or to your institution)
**Time frame: Since the initial planning of the work**			
**1**	All support for the present manuscript (e.g., funding, provision of study materials, medical writing, article processing charges, etc.) **No time limit for this item.**	☐ **None**<br>None	
			Click the tab key to add additional rows.
**Time frame: past 36 months**			
**2**	Grants or contracts from any entity (if not indicated in	☐ **None**<br>None	

		Name all entities with whom you have this relationship or indicate none (add rows as needed)	Specifications/Comments (e.g., if payments were made to you or to your institution)
	item #1 above).		
**3**	Royalties or licenses	☐ **None**<br>None	
**4**	Consulting fees	☐ **None**<br>None	
**5**	Payment or honoraria for lectures, presentations, speakers bureaus, manuscript writing or educational events	☐ **None**<br>None	
**6**	Payment for expert testimony	☐ **None**<br>None	
**7**	Support for attending meetings and/or travel	☐ **None**<br>None	
**8**	Patents planned, issued or pending	☐ **None**<br>None	
**9**	Participation on a Data Safety Monitoring Board or Advisory Board	☐ **None**<br>None	
**10**	Leadership	☐ **None**	

		Name all entities with whom you have this relationship or indicate none (add rows as needed)	Specifications/Comments (e.g., if payments were made to you or to your institution)
	or fiduciary role in other board, society, committee or advocacy group, paid or unpaid	None	
**11**	Stock or stock options	☐ **None**<br>None	
**12**	Receipt of equipment, materials, drugs, medical writing, gifts or other services	☐ **None**<br>None	
**13**	Other financial or non-financial interests	☐ **None**<br>None	

Please place an "X" next to the following statement to indicate your agreement:

☒ X

ICMJE DISCLOSURE FORM

Date: March 8, 2026	Click or tap to enter a date.
Your Name: Dilna Damodaran	Click or tap here to enter text.
Manuscript Title: Plasma Proteomic Signatures of Lipoprotein(a) and Cardiovascular Disease in Young Adults	Click or tap here to enter text.
Manuscript Number (if known): 204287-JCI-CRPH-1	Click or tap here to enter text.

In the interest of transparency, we ask you to disclose all relationships/activities/interests listed below that are related to the content of your manuscript. "Related" means any relation with for-profit or not-for-profit third parties whose interests may be affected by the content of the manuscript. Disclosure represents a commitment to transparency and does not necessarily indicate a bias. If you are in doubt about whether to list a relationship/activity/interest, it is preferable that you do so.

The author's relationships/activities/interests should be defined broadly. For example, if your manuscript pertains to the epidemiology of hypertension, you should declare all relationships with manufacturers of antihypertensive medication, even if that medication is not mentioned in the manuscript.

In item #1 below, report all support for the work reported in this manuscript without time limit. For all other items, the time frame for disclosure is the past 36 months.

		Name all entities with whom you have this relationship or indicate none (add rows as needed)	Specifications/Comments (e.g., if payments were made to you or to your institution)
**Time frame: Since the initial planning of the work**			
**1**	All support for the present manuscript (e.g., funding, provision of study materials, medical writing, article processing charges, etc.) **No time limit for this item.**	☐ **None**<br>None	
			Click the tab key to add additional rows.
**Time frame: past 36 months**			
**2**	Grants or contracts from any entity (if not indicated in	☐ **None**<br>None	

		Name all entities with whom you have this relationship or indicate none (add rows as needed)	Specifications/Comments (e.g., if payments were made to you or to your institution)
	item #1 above).		
3	Royalties or licenses	☐ **None**<br>None	
4	Consulting fees	☐ **None**<br>None	
5	Payment or honoraria for lectures, presentations, speakers bureaus, manuscript writing or educational events	☐ **None**<br>None	
6	Payment for expert testimony	☐ **None**<br>None	
7	Support for attending meetings and/or travel	☐ **None**<br>None	
8	Patents planned, issued or pending	☐ **None**<br>None	
9	Participation on a Data Safety Monitoring Board or Advisory Board	☐ **None**<br>None	
10	Leadership	☐ **None**	

		Name all entities with whom you have this relationship or indicate none (add rows as needed)	Specifications/Comments (e.g., if payments were made to you or to your institution)
	or fiduciary role in other board, society, committee or advocacy group, paid or unpaid	None	
**11**	Stock or stock options	☐ **None**<br>None	
**12**	Receipt of equipment, materials, drugs, medical writing, gifts or other services	☐ **None**<br>None	
**13**	Other financial or non-financial interests	☐ **None**<br>None	

Please place an "X" next to the following statement to indicate your agreement:

☒ X

ICMJE DISCLOSURE FORM

Date:<br>March 8, 2026	Click or tap to enter a date.
Your Name:<br>Brian T. Emmer	Click or tap here to enter text.
Manuscript Title:<br>Plasma Proteomic Signatures of Lipoprotein(a) and Cardiovascular Disease in Young Adults	Click or tap here to enter text.
Manuscript Number (if known):<br>204287-JCI-CRPH-1	Click or tap here to enter text.

In the interest of transparency, we ask you to disclose all relationships/activities/interests listed below that are related to the content of your manuscript. "Related" means any relation with for-profit or not-for-profit third parties whose interests may be affected by the content of the manuscript. Disclosure represents a commitment to transparency and does not necessarily indicate a bias. If you are in doubt about whether to list a relationship/activity/interest, it is preferable that you do so.

The author's relationships/activities/interests should be defined broadly. For example, if your manuscript pertains to the epidemiology of hypertension, you should declare all relationships with manufacturers of antihypertensive medication, even if that medication is not mentioned in the manuscript.

In item #1 below, report all support for the work reported in this manuscript without time limit. For all other items, the time frame for disclosure is the past 36 months.

		Name all entities with whom you have this relationship or indicate none (add rows as needed)	Specifications/Comments (e.g., if payments were made to you or to your institution)
**Time frame: Since the initial planning of the work**			
**1**	All support for the present manuscript (e.g., funding, provision of study materials, medical writing, article processing charges, etc.) **No time limit for this item.**	☐ **None**<br>NIH R01-HL167733, A. Alfred Taubman Medical Research Institute	
			Click the tab key to add additional rows.
**Time frame: past 36 months**			
2	Grants or contracts from any entity (if not indicated in	☐ **None**<br>None	

		Name all entities with whom you have this relationship or indicate none (add rows as needed)	Specifications/Comments (e.g., if payments were made to you or to your institution)
	item #1 above).		
3	Royalties or licenses	☐ **None**<br>None	
4	Consulting fees	☐ **None**<br>None	
5	Payment or honoraria for lectures, presentations, speakers bureaus, manuscript writing or educational events	☐ **None**<br>None	
6	Payment for expert testimony	☐ **None**<br>None	
7	Support for attending meetings and/or travel	☐ **None**<br>None	
8	Patents planned, issued or pending	☐ **None**<br>None	
9	Participation on a Data Safety Monitoring Board or Advisory Board	☐ **None**<br>None	
10	Leadership	☐ **None**	

		Name all entities with whom you have this relationship or indicate none (add rows as needed)	Specifications/Comments (e.g., if payments were made to you or to your institution)
	or fiduciary role in other board, society, committee or advocacy group, paid or unpaid	None	
**11**	Stock or stock options	☐ **None**<br>None	
**12**	Receipt of equipment, materials, drugs, medical writing, gifts or other services	☐ **None**<br>None	
**13**	Other financial or non-financial interests	☐ **None**<br>None	

Please place an "X" next to the following statement to indicate your agreement:

☒ X

ICMJE DISCLOSURE FORM

Date:<br>March 8, 2026	Click or tap to enter a date.
Your Name:<br>Kahraman Tanriverdi	Click or tap here to enter text.
Manuscript Title:<br>Plasma Proteomic Signatures of Lipoprotein(a) and Cardiovascular Disease in Young Adults	Click or tap here to enter text.
Manuscript Number (if known):<br>204287-JCI-CRPH-1	Click or tap here to enter text.

In the interest of transparency, we ask you to disclose all relationships/activities/interests listed below that are related to the content of your manuscript. "Related" means any relation with for-profit or not-for-profit third parties whose interests may be affected by the content of the manuscript. Disclosure represents a commitment to transparency and does not necessarily indicate a bias. If you are in doubt about whether to list a relationship/activity/interest, it is preferable that you do so.

The author's relationships/activities/interests should be defined broadly. For example, if your manuscript pertains to the epidemiology of hypertension, you should declare all relationships with manufacturers of antihypertensive medication, even if that medication is not mentioned in the manuscript.

In item #1 below, report all support for the work reported in this manuscript without time limit. For all other items, the time frame for disclosure is the past 36 months.

		Name all entities with whom you have this relationship or indicate none (add rows as needed)	Specifications/Comments (e.g., if payments were made to you or to your institution)
**Time frame: Since the initial planning of the work**			
**1**	All support for the present manuscript (e.g., funding, provision of study materials, medical writing, article processing charges, etc.) **No time limit for this item.**	☐ **None**<br>None	
			Click the tab key to add additional rows.
**Time frame: past 36 months**			
**2**	Grants or contracts from any entity (if not indicated in	☐ **None**<br>None	

		Name all entities with whom you have this relationship or indicate none (add rows as needed)	Specifications/Comments (e.g., if payments were made to you or to your institution)
	item #1 above).		
3	Royalties or licenses	☐ **None**<br>None	
4	Consulting fees	☐ **None**<br>None	
5	Payment or honoraria for lectures, presentations, speakers bureaus, manuscript writing or educational events	☐ **None**<br>None	
6	Payment for expert testimony	☐ **None**<br>None	
7	Support for attending meetings and/or travel	☐ **None**<br>None	
8	Patents planned, issued or pending	☐ **None**<br>None	
9	Participation on a Data Safety Monitoring Board or Advisory Board	☐ **None**<br>None	
10	Leadership	☐ **None**	

		Name all entities with whom you have this relationship or indicate none (add rows as needed)	Specifications/Comments (e.g., if payments were made to you or to your institution)
	or fiduciary role in other board, society, committee or advocacy group, paid or unpaid	None	
11	Stock or stock options	☐ **None**<br>None	
12	Receipt of equipment, materials, drugs, medical writing, gifts or other services	☐ **None**<br>None	
13	Other financial or non-financial interests	☐ **None**<br>None	

Please place an "X" next to the following statement to indicate your agreement:

☒ X

ICMJE DISCLOSURE FORM

Date:<br>March 8, 2026	Click or tap to enter a date.
Your Name:<br>Chirag J. Patel	Click or tap here to enter text.
Manuscript Title:<br>Plasma Proteomic Signatures of Lipoprotein(a) and Cardiovascular Disease in Young Adults	Click or tap here to enter text.
Manuscript Number (if known):<br>204287-JCI-CRPH-1	Click or tap here to enter text.

In the interest of transparency, we ask you to disclose all relationships/activities/interests listed below that are related to the content of your manuscript. "Related" means any relation with for-profit or not-for-profit third parties whose interests may be affected by the content of the manuscript. Disclosure represents a commitment to transparency and does not necessarily indicate a bias. If you are in doubt about whether to list a relationship/activity/interest, it is preferable that you do so.

The author's relationships/activities/interests should be defined broadly. For example, if your manuscript pertains to the epidemiology of hypertension, you should declare all relationships with manufacturers of antihypertensive medication, even if that medication is not mentioned in the manuscript.

In item #1 below, report all support for the work reported in this manuscript without time limit. For all other items, the time frame for disclosure is the past 36 months.

		**Name all entities with whom you have this relationship or indicate none (add rows as needed)**	**Specifications/Comments (e.g., if payments were made to you or to your institution)**
**Time frame: Since the initial planning of the work**			
**1**	All support for the present manuscript (e.g., funding, provision of study materials, medical writing, article processing charges, etc.) **No time limit for this item.**	☐ **None**<br>None	
			Click the tab key to add additional rows.
**Time frame: past 36 months**			
**2**	Grants or contracts from any entity (if not indicated in	☐ **None**<br>None	

		Name all entities with whom you have this relationship or indicate none (add rows as needed)	Specifications/Comments (e.g., if payments were made to you or to your institution)
	item #1 above).		
3	Royalties or licenses	☐ **None**<br>None	
4	Consulting fees	☐ **None**<br>None	
5	Payment or honoraria for lectures, presentations, speakers bureaus, manuscript writing or educational events	☐ **None**<br>None	
6	Payment for expert testimony	☐ **None**<br>None	
7	Support for attending meetings and/or travel	☐ **None**<br>None	
8	Patents planned, issued or pending	☐ **None**<br>None	
9	Participation on a Data Safety Monitoring Board or Advisory Board	☐ **None**<br>None	
10	Leadership	☐ **None**	

		Name all entities with whom you have this relationship or indicate none (add rows as needed)	Specifications/Comments (e.g., if payments were made to you or to your institution)
	or fiduciary role in other board, society, committee or advocacy group, paid or unpaid	None	
11	Stock or stock options	☐ **None**<br>None	
12	Receipt of equipment, materials, drugs, medical writing, gifts or other services	☐ **None**<br>None	
13	Other financial or non-financial interests	☐ **None**<br>None	

Please place an "X" next to the following statement to indicate your agreement:

☒ X

ICMJE DISCLOSURE FORM

Date: March 8, 2026	Click or tap to enter a date.
Your Name: John T. Wilkins	Click or tap here to enter text.
Manuscript Title: Plasma Proteomic Signatures of Lipoprotein(a) and Cardiovascular Disease in Young Adults	Click or tap here to enter text.
Manuscript Number (if known): 204287-JCI-CRPH-1	Click or tap here to enter text.

In the interest of transparency, we ask you to disclose all relationships/activities/interests listed below that are related to the content of your manuscript. "Related" means any relation with for-profit or not-for-profit third parties whose interests may be affected by the content of the manuscript. Disclosure represents a commitment to transparency and does not necessarily indicate a bias. If you are in doubt about whether to list a relationship/activity/interest, it is preferable that you do so.

The author's relationships/activities/interests should be defined broadly. For example, if your manuscript pertains to the epidemiology of hypertension, you should declare all relationships with manufacturers of antihypertensive medication, even if that medication is not mentioned in the manuscript.

In item #1 below, report all support for the work reported in this manuscript without time limit. For all other items, the time frame for disclosure is the past 36 months.

		Name all entities with whom you have this relationship or indicate none (add rows as needed)	Specifications/Comments (e.g., if payments were made to you or to your institution)
**Time frame: Since the initial planning of the work**			
**1**	All support for the present manuscript (e.g., funding, provision of study materials, medical writing, article processing charges, etc.) **No time limit for this item.**	☐ **None**<br>None	
			Click the tab key to add additional rows.
**Time frame: past 36 months**			
**2**	Grants or contracts from any entity (if not indicated in	☐ **None**<br>None	

		Name all entities with whom you have this relationship or indicate none (add rows as needed)	Specifications/Comments (e.g., if payments were made to you or to your institution)
	item #1 above).		
3	Royalties or licenses	☐ **None**<br>None	
4	Consulting fees	☐ **None**<br>None	
5	Payment or honoraria for lectures, presentations, speakers bureaus, manuscript writing or educational events	☐ **None**<br>None	
6	Payment for expert testimony	☐ **None**<br>None	
7	Support for attending meetings and/or travel	☐ **None**<br>None	
8	Patents planned, issued or pending	☐ **None**<br>None	
9	Participation on a Data Safety Monitoring Board or Advisory Board	☐ **None**<br>None	
10	Leadership	☐ **None**	

		Name all entities with whom you have this relationship or indicate none (add rows as needed)	Specifications/Comments (e.g., if payments were made to you or to your institution)
	or fiduciary role in other board, society, committee or advocacy group, paid or unpaid	None	
**11**	Stock or stock options	☐ **None**<br>None	
**12**	Receipt of equipment, materials, drugs, medical writing, gifts or other services	☐ **None**<br>None	
**13**	Other financial or non-financial interests	☐ **None**<br>None	

Please place an "X" next to the following statement to indicate your agreement:

☒ X

ICMJE DISCLOSURE FORM

Date:<br>March 8, 2026	Click or tap to enter a date.
Your Name:<br>Mark A. Sarzynski	Click or tap here to enter text.
Manuscript Title:<br>Plasma Proteomic Signatures of Lipoprotein(a) and Cardiovascular Disease in Young Adults	Click or tap here to enter text.
Manuscript Number (if known):<br>204287-JCI-CRPH-1	Click or tap here to enter text.

In the interest of transparency, we ask you to disclose all relationships/activities/interests listed below that are related to the content of your manuscript. "Related" means any relation with for-profit or not-for-profit third parties whose interests may be affected by the content of the manuscript. Disclosure represents a commitment to transparency and does not necessarily indicate a bias. If you are in doubt about whether to list a relationship/activity/interest, it is preferable that you do so.

The author's relationships/activities/interests should be defined broadly. For example, if your manuscript pertains to the epidemiology of hypertension, you should declare all relationships with manufacturers of antihypertensive medication, even if that medication is not mentioned in the manuscript.

In item #1 below, report all support for the work reported in this manuscript without time limit. For all other items, the time frame for disclosure is the past 36 months.

		Name all entities with whom you have this relationship or indicate none (add rows as needed)	Specifications/Comments (e.g., if payments were made to you or to your institution)
**Time frame: Since the initial planning of the work**			
**1**	All support for the present manuscript (e.g., funding, provision of study materials, medical writing, article processing charges, etc.)<br>**No time limit for this item.**	☐ **None**<br>National Institute of Nursing Research R01NR019628	
			Click the tab key to add additional rows.
**Time frame: past 36 months**			
**2**	Grants or contracts from any entity (if not indicated in	☐ **None**<br>None	

		Name all entities with whom you have this relationship or indicate none (add rows as needed)	Specifications/Comments (e.g., if payments were made to you or to your institution)
	item #1 above).		
3	Royalties or licenses	☐ **None**<br>None	
4	Consulting fees	☐ **None**<br>None	
5	Payment or honoraria for lectures, presentations, speakers bureaus, manuscript writing or educational events	☐ **None**<br>None	
6	Payment for expert testimony	☐ **None**<br>Genetic Direction	
7	Support for attending meetings and/or travel	☐ **None**<br>None	
8	Patents planned, issued or pending	☐ **None**<br>None	
9	Participation on a Data Safety Monitoring Board or Advisory Board	☐ **None**<br>None	
10	Leadership	☐ **None**	

		Name all entities with whom you have this relationship or indicate none (add rows as needed)	Specifications/Comments (e.g., if payments were made to you or to your institution)
	or fiduciary role in other board, society, committee or advocacy group, paid or unpaid	None	
**11**	Stock or stock options	☐ **None**<br>Scientific Advisory Board for Elysium Health	
**12**	Receipt of equipment, materials, drugs, medical writing, gifts or other services	☐ **None**<br>None	
**13**	Other financial or non-financial interests	☐ **None**<br>None	

Please place an "X" next to the following statement to indicate your agreement:

☒ X

ICMJE DISCLOSURE FORM

Date:<br>March 8, 2026	Click or tap to enter a date.
Your Name:<br>Vera Bittner	Click or tap here to enter text.
Manuscript Title:<br>Plasma Proteomic Signatures of Lipoprotein(a) and Cardiovascular Disease in Young Adults	Click or tap here to enter text.
Manuscript Number (if known):<br>204287-JCI-CRPH-1	Click or tap here to enter text.

In the interest of transparency, we ask you to disclose all relationships/activities/interests listed below that are related to the content of your manuscript. "Related" means any relation with for-profit or not-for-profit third parties whose interests may be affected by the content of the manuscript. Disclosure represents a commitment to transparency and does not necessarily indicate a bias. If you are in doubt about whether to list a relationship/activity/interest, it is preferable that you do so.

The author's relationships/activities/interests should be defined broadly. For example, if your manuscript pertains to the epidemiology of hypertension, you should declare all relationships with manufacturers of antihypertensive medication, even if that medication is not mentioned in the manuscript.

In item #1 below, report all support for the work reported in this manuscript without time limit. For all other items, the time frame for disclosure is the past 36 months.

		Name all entities with whom you have this relationship or indicate none (add rows as needed)	Specifications/Comments (e.g., if payments were made to you or to your institution)
**Time frame: Since the initial planning of the work**			
**1**	All support for the present manuscript (e.g., funding, provision of study materials, medical writing, article processing charges, etc.) **No time limit for this item.**	☐ **None**<br>None	
			Click the tab key to add additional rows.
**Time frame: past 36 months**			
**2**	Grants or contracts from any entity (if not indicated in	☐ **None**<br>NHLBI via Wake Forest University, Amgen, Dalcor, Esperion, Novartis, Sanofi and Regeneron	

		Name all entities with whom you have this relationship or indicate none (add rows as needed)	Specifications/Comments (e.g., if payments were made to you or to your institution)
	item #1 above).		
**3**	Royalties or licenses	☐ **None**<br>None	
**4**	Consulting fees	☐ **None**<br>None	
**5**	Payment or honoraria for lectures, presentations, speakers bureaus, manuscript writing or educational events	☐ **None**<br>None	
**6**	Payment for expert testimony	☐ **None**<br>None	
**7**	Support for attending meetings and/or travel	☐ **None**<br>American College of Cardiology, American Heart Association, National Lipid Association, New Amsterdam Pharma	
**8**	Patents planned, issued or pending	☐ **None**<br>None	
**9**	Participation on a Data Safety Monitoring Board or Advisory Board	☐ **None**<br>None	
**10**	Leadership	☐ **None**	

		Name all entities with whom you have this relationship or indicate none (add rows as needed)	Specifications/Comments (e.g., if payments were made to you or to your institution)
	or fiduciary role in other board, society, committee or advocacy group, paid or unpaid	None	
**11**	Stock or stock options	☐ **None**<br>NIH-NIA, Eli Lilly, Verve Therapeutics	
**12**	Receipt of equipment, materials, drugs, medical writing, gifts or other services	☐ **None**<br>None	
**13**	Other financial or non-financial interests	☐ **None**<br>None	

Please place an "X" next to the following statement to indicate your agreement:

☒ X

ICMJE DISCLOSURE FORM

Date:<br>March 8, 2026	Click or tap to enter a date.
Your Name:<br>Ron Do	Click or tap here to enter text.
Manuscript Title:<br>Plasma Proteomic Signatures of Lipoprotein(a) and Cardiovascular Disease in Young Adults	Click or tap here to enter text.
Manuscript Number (if known):<br>204287-JCI-CRPH-1	Click or tap here to enter text.

In the interest of transparency, we ask you to disclose all relationships/activities/interests listed below that are related to the content of your manuscript. "Related" means any relation with for-profit or not-for-profit third parties whose interests may be affected by the content of the manuscript. Disclosure represents a commitment to transparency and does not necessarily indicate a bias. If you are in doubt about whether to list a relationship/activity/interest, it is preferable that you do so.

The author's relationships/activities/interests should be defined broadly. For example, if your manuscript pertains to the epidemiology of hypertension, you should declare all relationships with manufacturers of antihypertensive medication, even if that medication is not mentioned in the manuscript.

In item #1 below, report all support for the work reported in this manuscript without time limit. For all other items, the time frame for disclosure is the past 36 months.

		Name all entities with whom you have this relationship or indicate none (add rows as needed)	Specifications/Comments (e.g., if payments were made to you or to your institution)
**Time frame: Since the initial planning of the work**			
**1**	All support for the present manuscript (e.g., funding, provision of study materials, medical writing, article processing charges, etc.) **No time limit for this item.**	☐ **None**<br>None	
			Click the tab key to add additional rows.
**Time frame: past 36 months**			
2	Grants or contracts from any entity (if not indicated in	☐ **None**<br>NIH/NIGMS R35-GM124836	

		Name all entities with whom you have this relationship or indicate none (add rows as needed)	Specifications/Comments (e.g., if payments were made to you or to your institution)
	item #1 above).		
3	Royalties or licenses	☐ **None**<br>None	
4	Consulting fees	☐ **None**<br>None	
5	Payment or honoraria for lectures, presentations, speakers bureaus, manuscript writing or educational events	☐ **None**<br>None	
6	Payment for expert testimony	☐ **None**<br>None	
7	Support for attending meetings and/or travel	☐ **None**<br>None	
8	Patents planned, issued or pending	☐ **None**<br>None	
9	Participation on a Data Safety Monitoring Board or Advisory Board	☐ **None**<br>None	
10	Leadership	☐ **None**	

		Name all entities with whom you have this relationship or indicate none (add rows as needed)	Specifications/Comments (e.g., if payments were made to you or to your institution)
	or fiduciary role in other board, society, committee or advocacy group, paid or unpaid	None	
**11**	Stock or stock options	☐ **None**<br>None	
**12**	Receipt of equipment, materials, drugs, medical writing, gifts or other services	☐ **None**<br>None	
**13**	Other financial or non-financial interests	☐ **None**<br>Scientific cofounder, consultant, and equity holder (pending) for Pensieve Health; consultant for Variant Bio (all unrelated to this work)	

Please place an "X" next to the following statement to indicate your agreement:

☒ X

ICMJE DISCLOSURE FORM

Date:<br>March 8, 2026	Click or tap to enter a date.
Your Name:<br>Supriya Shore	Click or tap here to enter text.
Manuscript Title:<br>Plasma Proteomic Signatures of Lipoprotein(a) and Cardiovascular Disease in Young Adults	Click or tap here to enter text.
Manuscript Number (if known):<br>204287-JCI-CRPH-1	Click or tap here to enter text.

In the interest of transparency, we ask you to disclose all relationships/activities/interests listed below that are related to the content of your manuscript. "Related" means any relation with for-profit or not-for-profit third parties whose interests may be affected by the content of the manuscript. Disclosure represents a commitment to transparency and does not necessarily indicate a bias. If you are in doubt about whether to list a relationship/activity/interest, it is preferable that you do so.

The author's relationships/activities/interests should be defined broadly. For example, if your manuscript pertains to the epidemiology of hypertension, you should declare all relationships with manufacturers of antihypertensive medication, even if that medication is not mentioned in the manuscript.

In item #1 below, report all support for the work reported in this manuscript without time limit. For all other items, the time frame for disclosure is the past 36 months.

		Name all entities with whom you have this relationship or indicate none (add rows as needed)	Specifications/Comments (e.g., if payments were made to you or to your institution)
**Time frame: Since the initial planning of the work**			
**1**	All support for the present manuscript (e.g., funding, provision of study materials, medical writing, article processing charges, etc.)<br>**No time limit for this item.**	☐ **None**<br>None	
			Click the tab key to add additional rows.
**Time frame: past 36 months**			
**2**	Grants or contracts from any entity (if not indicated in	☐ **None**<br>None	

		Name all entities with whom you have this relationship or indicate none (add rows as needed)	Specifications/Comments (e.g., if payments were made to you or to your institution)
	item #1 above).		
3	Royalties or licenses	☐ **None**<br>None	
4	Consulting fees	☐ **None**<br>None	
5	Payment or honoraria for lectures, presentations, speakers bureaus, manuscript writing or educational events	☐ **None**<br>None	
6	Payment for expert testimony	☐ **None**<br>None	
7	Support for attending meetings and/or travel	☐ **None**<br>None	
8	Patents planned, issued or pending	☐ **None**<br>None	
9	Participation on a Data Safety Monitoring Board or Advisory Board	☐ **None**<br>None	
10	Leadership	☐ **None**	

		Name all entities with whom you have this relationship or indicate none (add rows as needed)	Specifications/Comments (e.g., if payments were made to you or to your institution)
	or fiduciary role in other board, society, committee or advocacy group, paid or unpaid	None	
**11**	Stock or stock options	☐ **None**<br>None	
**12**	Receipt of equipment, materials, drugs, medical writing, gifts or other services	☐ **None**<br>None	
**13**	Other financial or non-financial interests	☐ **None**<br>None	

Please place an "X" next to the following statement to indicate your agreement:

☒ X

ICMJE DISCLOSURE FORM

Date:<br>March 8, 2026	Click or tap to enter a date.
Your Name:<br>Jane E. Freedman	Click or tap here to enter text.
Manuscript Title:<br>Plasma Proteomic Signatures of Lipoprotein(a) and Cardiovascular Disease in Young Adults	Click or tap here to enter text.
Manuscript Number (if known):<br>204287-JCI-CRPH-1	Click or tap here to enter text.

In the interest of transparency, we ask you to disclose all relationships/activities/interests listed below that are related to the content of your manuscript. "Related" means any relation with for-profit or not-for-profit third parties whose interests may be affected by the content of the manuscript. Disclosure represents a commitment to transparency and does not necessarily indicate a bias. If you are in doubt about whether to list a relationship/activity/interest, it is preferable that you do so.

The author's relationships/activities/interests should be defined broadly. For example, if your manuscript pertains to the epidemiology of hypertension, you should declare all relationships with manufacturers of antihypertensive medication, even if that medication is not mentioned in the manuscript.

In item #1 below, report all support for the work reported in this manuscript without time limit. For all other items, the time frame for disclosure is the past 36 months.

		Name all entities with whom you have this relationship or indicate none (add rows as needed)	Specifications/Comments (e.g., if payments were made to you or to your institution)
**Time frame: Since the initial planning of the work**			
**1**	All support for the present manuscript (e.g., funding, provision of study materials, medical writing, article processing charges, etc.) **No time limit for this item.**	☐ **None**<br>None	
			Click the tab key to add additional rows.
**Time frame: past 36 months**			
**2**	Grants or contracts from any entity (if not indicated in	☐ **None**<br>None	

<table>
<tr><th colspan="2"></th><th>Name all entities with whom you have this relationship or indicate none (add rows as needed)</th><th>Specifications/Comments (e.g., if payments were made to you or to your institution)</th></tr>
<tr><td></td><td>item #1 above).</td><td colspan="2"></td></tr>
<tr><td>3</td><td>Royalties or licenses</td><td colspan="2">☐ None
None</td></tr>
<tr><td>4</td><td>Consulting fees</td><td colspan="2">☐ None
None</td></tr>
<tr><td>5</td><td>Payment or honoraria for lectures, presentations, speakers bureaus, manuscript writing or educational events</td><td colspan="2">☐ None
None</td></tr>
<tr><td>6</td><td>Payment for expert testimony</td><td colspan="2">☐ None
None</td></tr>
<tr><td>7</td><td>Support for attending meetings and/or travel</td><td colspan="2">☐ None
None</td></tr>
<tr><td>8</td><td>Patents planned, issued or pending</td><td colspan="2">☐ None
None</td></tr>
<tr><td>9</td><td>Participation on a Data Safety Monitoring Board or Advisory Board</td><td colspan="2">☐ None
None</td></tr>
<tr><td>10</td><td>Leadership</td><td colspan="2">☐ None</td></tr>
</table>

		Name all entities with whom you have this relationship or indicate none (add rows as needed)	Specifications/Comments (e.g., if payments were made to you or to your institution)
	or fiduciary role in other board, society, committee or advocacy group, paid or unpaid	None	
11	Stock or stock options	☐ **None**<br>None	
12	Receipt of equipment, materials, drugs, medical writing, gifts or other services	☐ **None**<br>None	
13	Other financial or non-financial interests	☐ **None**<br>None	

Please place an "X" next to the following statement to indicate your agreement:

☒ X

ICMJE DISCLOSURE FORM

Date: March 8, 2026	Click or tap to enter a date.
Your Name: David R. Jacobs, Jr.	Click or tap here to enter text.
Manuscript Title: Plasma Proteomic Signatures of Lipoprotein(a) and Cardiovascular Disease in Young Adults	Click or tap here to enter text.
Manuscript Number (if known): 204287-JCI-CRPH-1	Click or tap here to enter text.

In the interest of transparency, we ask you to disclose all relationships/activities/interests listed below that are related to the content of your manuscript. "Related" means any relation with for-profit or not-for-profit third parties whose interests may be affected by the content of the manuscript. Disclosure represents a commitment to transparency and does not necessarily indicate a bias. If you are in doubt about whether to list a relationship/activity/interest, it is preferable that you do so.

The author's relationships/activities/interests should be defined broadly. For example, if your manuscript pertains to the epidemiology of hypertension, you should declare all relationships with manufacturers of antihypertensive medication, even if that medication is not mentioned in the manuscript.

In item #1 below, report all support for the work reported in this manuscript without time limit. For all other items, the time frame for disclosure is the past 36 months.

		Name all entities with whom you have this relationship or indicate none (add rows as needed)	Specifications/Comments (e.g., if payments were made to you or to your institution)
**Time frame: Since the initial planning of the work**			
**1**	All support for the present manuscript (e.g., funding, provision of study materials, medical writing, article processing charges, etc.) **No time limit for this item.**	☐ **None**<br>None	
			Click the tab key to add additional rows.
**Time frame: past 36 months**			
**2**	Grants or contracts from any entity (if not indicated in	☐ **None**<br>None	

		Name all entities with whom you have this relationship or indicate none (add rows as needed)	Specifications/Comments (e.g., if payments were made to you or to your institution)
	item #1 above).		
**3**	Royalties or licenses	☐ **None**<br>None	
**4**	Consulting fees	☐ **None**<br>None	
**5**	Payment or honoraria for lectures, presentations, speakers bureaus, manuscript writing or educational events	☐ **None**<br>None	
**6**	Payment for expert testimony	☐ **None**<br>None	
**7**	Support for attending meetings and/or travel	☐ **None**<br>None	
**8**	Patents planned, issued or pending	☐ **None**<br>None	
**9**	Participation on a Data Safety Monitoring Board or Advisory Board	☐ **None**<br>None	
**10**	Leadership	☐ **None**	

		Name all entities with whom you have this relationship or indicate none (add rows as needed)	Specifications/Comments (e.g., if payments were made to you or to your institution)
	or fiduciary role in other board, society, committee or advocacy group, paid or unpaid	None	
**11**	Stock or stock options	☐ **None**<br>None	
**12**	Receipt of equipment, materials, drugs, medical writing, gifts or other services	☐ **None**<br>None	
**13**	Other financial or non-financial interests	☐ **None**<br>None	

Please place an "X" next to the following statement to indicate your agreement:

☒ X

ICMJE DISCLOSURE FORM

Date:<br>March 8, 2026	Click or tap to enter a date.
Your Name:<br>Bharat Thyagarajan	Click or tap here to enter text.
Manuscript Title:<br>Plasma Proteomic Signatures of Lipoprotein(a) and Cardiovascular Disease in Young Adults	Click or tap here to enter text.
Manuscript Number (if known):<br>204287-JCI-CRPH-1	Click or tap here to enter text.

In the interest of transparency, we ask you to disclose all relationships/activities/interests listed below that are related to the content of your manuscript. "Related" means any relation with for-profit or not-for-profit third parties whose interests may be affected by the content of the manuscript. Disclosure represents a commitment to transparency and does not necessarily indicate a bias. If you are in doubt about whether to list a relationship/activity/interest, it is preferable that you do so.

The author's relationships/activities/interests should be defined broadly. For example, if your manuscript pertains to the epidemiology of hypertension, you should declare all relationships with manufacturers of antihypertensive medication, even if that medication is not mentioned in the manuscript.

In item #1 below, report all support for the work reported in this manuscript without time limit. For all other items, the time frame for disclosure is the past 36 months.

		Name all entities with whom you have this relationship or indicate none (add rows as needed)	Specifications/Comments (e.g., if payments were made to you or to your institution)
**Time frame: Since the initial planning of the work**			
**1**	All support for the present manuscript (e.g., funding, provision of study materials, medical writing, article processing charges, etc.) **No time limit for this item.**	☐ **None**<br>None	
			Click the tab key to add additional rows.
**Time frame: past 36 months**			
**2**	Grants or contracts from any entity (if not indicated in	☐ **None**<br>None	

		Name all entities with whom you have this relationship or indicate none (add rows as needed)	Specifications/Comments (e.g., if payments were made to you or to your institution)
	item #1 above).		
3	Royalties or licenses	☐ **None**<br>None	
4	Consulting fees	☐ **None**<br>None	
5	Payment or honoraria for lectures, presentations, speakers bureaus, manuscript writing or educational events	☐ **None**<br>None	
6	Payment for expert testimony	☐ **None**<br>None	
7	Support for attending meetings and/or travel	☐ **None**<br>None	
8	Patents planned, issued or pending	☐ **None**<br>None	
9	Participation on a Data Safety Monitoring Board or Advisory Board	☐ **None**<br>None	
10	Leadership	☐ **None**	

		Name all entities with whom you have this relationship or indicate none (add rows as needed)	Specifications/Comments (e.g., if payments were made to you or to your institution)
	or fiduciary role in other board, society, committee or advocacy group, paid or unpaid	None	
11	Stock or stock options	☐ **None**<br>None	
12	Receipt of equipment, materials, drugs, medical writing, gifts or other services	☐ **None**<br>None	
13	Other financial or non-financial interests	☐ **None**<br>None	

Please place an "X" next to the following statement to indicate your agreement:

☒ X

ICMJE DISCLOSURE FORM

Date: March 8, 2026	Click or tap to enter a date.
Your Name: Clary Clish	Click or tap here to enter text.
Manuscript Title: Plasma Proteomic Signatures of Lipoprotein(a) and Cardiovascular Disease in Young Adults	Click or tap here to enter text.
Manuscript Number (if known): 204287-JCI-CRPH-1	Click or tap here to enter text.

In the interest of transparency, we ask you to disclose all relationships/activities/interests listed below that are related to the content of your manuscript. "Related" means any relation with for-profit or not-for-profit third parties whose interests may be affected by the content of the manuscript. Disclosure represents a commitment to transparency and does not necessarily indicate a bias. If you are in doubt about whether to list a relationship/activity/interest, it is preferable that you do so.

The author's relationships/activities/interests should be defined broadly. For example, if your manuscript pertains to the epidemiology of hypertension, you should declare all relationships with manufacturers of antihypertensive medication, even if that medication is not mentioned in the manuscript.

In item #1 below, report all support for the work reported in this manuscript without time limit. For all other items, the time frame for disclosure is the past 36 months.

		Name all entities with whom you have this relationship or indicate none (add rows as needed)	Specifications/Comments (e.g., if payments were made to you or to your institution)
**Time frame: Since the initial planning of the work**			
**1**	All support for the present manuscript (e.g., funding, provision of study materials, medical writing, article processing charges, etc.) **No time limit for this item.**	☐ **None**<br>None	
			Click the tab key to add additional rows.
**Time frame: past 36 months**			
**2**	Grants or contracts from any entity (if not indicated in	☐ **None**<br>None	

		Name all entities with whom you have this relationship or indicate none (add rows as needed)	Specifications/Comments (e.g., if payments were made to you or to your institution)
	item #1 above).		
3	Royalties or licenses	☐ **None**<br>None	
4	Consulting fees	☐ **None**<br>None	
5	Payment or honoraria for lectures, presentations, speakers bureaus, manuscript writing or educational events	☐ **None**<br>None	
6	Payment for expert testimony	☐ **None**<br>None	
7	Support for attending meetings and/or travel	☐ **None**<br>None	
8	Patents planned, issued or pending	☐ **None**<br>None	
9	Participation on a Data Safety Monitoring Board or Advisory Board	☐ **None**<br>None	
10	Leadership	☐ **None**	

		Name all entities with whom you have this relationship or indicate none (add rows as needed)	Specifications/Comments (e.g., if payments were made to you or to your institution)
	or fiduciary role in other board, society, committee or advocacy group, paid or unpaid	None	
**11**	Stock or stock options	☐ **None**<br>None	
**12**	Receipt of equipment, materials, drugs, medical writing, gifts or other services	☐ **None**<br>None	
**13**	Other financial or non-financial interests	☐ **None**<br>None	

Please place an "X" next to the following statement to indicate your agreement:

☒ X

ICMJE DISCLOSURE FORM

Date:<br>March 8, 2026	Click or tap to enter a date.
Your Name:<br>Marios K. Georgakis	Click or tap here to enter text.
Manuscript Title:<br>Plasma Proteomic Signatures of Lipoprotein(a) and Cardiovascular Disease in Young Adults	Click or tap here to enter text.
Manuscript Number (if known):<br>204287-JCI-CRPH-1	Click or tap here to enter text.

In the interest of transparency, we ask you to disclose all relationships/activities/interests listed below that are related to the content of your manuscript. "Related" means any relation with for-profit or not-for-profit third parties whose interests may be affected by the content of the manuscript. Disclosure represents a commitment to transparency and does not necessarily indicate a bias. If you are in doubt about whether to list a relationship/activity/interest, it is preferable that you do so.

The author's relationships/activities/interests should be defined broadly. For example, if your manuscript pertains to the epidemiology of hypertension, you should declare all relationships with manufacturers of antihypertensive medication, even if that medication is not mentioned in the manuscript.

In item #1 below, report all support for the work reported in this manuscript without time limit. For all other items, the time frame for disclosure is the past 36 months.

		Name all entities with whom you have this relationship or indicate none (add rows as needed)	Specifications/Comments (e.g., if payments were made to you or to your institution)
**Time frame: Since the initial planning of the work**			
**1**	All support for the present manuscript (e.g., funding, provision of study materials, medical writing, article processing charges, etc.) **No time limit for this item.**	☐ **None**<br>None	
			Click the tab key to add additional rows.
**Time frame: past 36 months**			
**2**	Grants or contracts from any entity (if not indicated in	☐ **None**<br>None	

		Name all entities with whom you have this relationship or indicate none (add rows as needed)	Specifications/Comments (e.g., if payments were made to you or to your institution)
	item #1 above).		
**3**	Royalties or licenses	☐ **None**<br>None	
**4**	Consulting fees	☐ **None**<br>None	
**5**	Payment or honoraria for lectures, presentations, speakers bureaus, manuscript writing or educational events	☐ **None**<br>None	
**6**	Payment for expert testimony	☐ **None**<br>None	
**7**	Support for attending meetings and/or travel	☐ **None**<br>None	
**8**	Patents planned, issued or pending	☐ **None**<br>None	
**9**	Participation on a Data Safety Monitoring Board or Advisory Board	☐ **None**<br>None	
**10**	Leadership	☐ **None**	

		Name all entities with whom you have this relationship or indicate none (add rows as needed)	Specifications/Comments (e.g., if payments were made to you or to your institution)
	or fiduciary role in other board, society, committee or advocacy group, paid or unpaid	None	
**11**	Stock or stock options	☐ **None**<br>None	
**12**	Receipt of equipment, materials, drugs, medical writing, gifts or other services	☐ **None**<br>None	
**13**	Other financial or non-financial interests	☐ **None**<br>None	

Please place an "X" next to the following statement to indicate your agreement:

☒ X

ICMJE DISCLOSURE FORM

Date: March 8, 2026	Click or tap to enter a date.
Your Name: Eric J. Brandt	Click or tap here to enter text.
Manuscript Title: Plasma Proteomic Signatures of Lipoprotein(a) and Cardiovascular Disease in Young Adults	Click or tap here to enter text.
Manuscript Number (if known): 204287-JCI-CRPH-1	Click or tap here to enter text.

In the interest of transparency, we ask you to disclose all relationships/activities/interests listed below that are related to the content of your manuscript. "Related" means any relation with for-profit or not-for-profit third parties whose interests may be affected by the content of the manuscript. Disclosure represents a commitment to transparency and does not necessarily indicate a bias. If you are in doubt about whether to list a relationship/activity/interest, it is preferable that you do so.

The author's relationships/activities/interests should be defined broadly. For example, if your manuscript pertains to the epidemiology of hypertension, you should declare all relationships with manufacturers of antihypertensive medication, even if that medication is not mentioned in the manuscript.

In item #1 below, report all support for the work reported in this manuscript without time limit. For all other items, the time frame for disclosure is the past 36 months.

		Name all entities with whom you have this relationship or indicate none (add rows as needed)	Specifications/Comments (e.g., if payments were made to you or to your institution)
**Time frame: Since the initial planning of the work**			
**1**	All support for the present manuscript (e.g., funding, provision of study materials, medical writing, article processing charges, etc.) **No time limit for this item.**	☐ **None**<br>NIH K23MD017253, Blue Cross Blue Shield of Michigan Foundation	
			Click the tab key to add additional rows.
**Time frame: past 36 months**			
**2**	Grants or contracts from any entity (if not indicated in	☐ **None**<br>None	

		Name all entities with whom you have this relationship or indicate none (add rows as needed)	Specifications/Comments (e.g., if payments were made to you or to your institution)
	item #1 above).		
3	Royalties or licenses	☐ **None**<br>None	
4	Consulting fees	☐ **None**<br>None	
5	Payment or honoraria for lectures, presentations, speakers bureaus, manuscript writing or educational events	☐ **None**<br>None	
6	Payment for expert testimony	☐ **None**<br>New Amsterdam Pharmaceuticals	
7	Support for attending meetings and/or travel	☐ **None**<br>None	
8	Patents planned, issued or pending	☐ **None**<br>None	
9	Participation on a Data Safety Monitoring Board or Advisory Board	☐ **None**<br>None	
10	Leadership	☐ **None**	

		Name all entities with whom you have this relationship or indicate none (add rows as needed)	Specifications/Comments (e.g., if payments were made to you or to your institution)
	or fiduciary role in other board, society, committee or advocacy group, paid or unpaid	None	
**11**	Stock or stock options	☐ **None**<br>None	
**12**	Receipt of equipment, materials, drugs, medical writing, gifts or other services	☐ **None**<br>None	
**13**	Other financial or non-financial interests	☐ **None**<br>None	

Please place an "X" next to the following statement to indicate your agreement:

☒ X

ICMJE DISCLOSURE FORM

Date:<br>March 8, 2026	Click or tap to enter a date.
Your Name:<br>Venkatesh L. Murthy	Click or tap here to enter text.
Manuscript Title:<br>Plasma Proteomic Signatures of Lipoprotein(a) and Cardiovascular Disease in Young Adults	Click or tap here to enter text.
Manuscript Number (if known):<br>204287-JCI-CRPH-1	Click or tap here to enter text.

In the interest of transparency, we ask you to disclose all relationships/activities/interests listed below that are related to the content of your manuscript. "Related" means any relation with for-profit or not-for-profit third parties whose interests may be affected by the content of the manuscript. Disclosure represents a commitment to transparency and does not necessarily indicate a bias. If you are in doubt about whether to list a relationship/activity/interest, it is preferable that you do so.

The author's relationships/activities/interests should be defined broadly. For example, if your manuscript pertains to the epidemiology of hypertension, you should declare all relationships with manufacturers of antihypertensive medication, even if that medication is not mentioned in the manuscript.

In item #1 below, report all support for the work reported in this manuscript without time limit. For all other items, the time frame for disclosure is the past 36 months.

		Name all entities with whom you have this relationship or indicate none (add rows as needed)	Specifications/Comments (e.g., if payments were made to you or to your institution)
**Time frame: Since the initial planning of the work**			
**1**	All support for the present manuscript (e.g., funding, provision of study materials, medical writing, article processing charges, etc.) **No time limit for this item.**	☐ **None**<br>NIDDK U01DK123013-03, NIA R01 AG059729, NHLBI R01 HL136685, AHA Strategically Focused Research Network	
			Click the tab key to add additional rows.
**Time frame: past 36 months**			
**2**	Grants or contracts from any entity (if not indicated in	☐ **None**<br>Siemens Medical Imaging, Jubilant Draximage, Credence Management Solutions	

		Name all entities with whom you have this relationship or indicate none (add rows as needed)	Specifications/Comments (e.g., if payments were made to you or to your institution)
	item #1 above).		
**3**	Royalties or licenses	☐ **None**<br>None	
**4**	Consulting fees	☐ **None**<br>None	
**5**	Payment or honoraria for lectures, presentations, speakers bureaus, manuscript writing or educational events	☐ **None**<br>None	
**6**	Payment for expert testimony	☐ **None**<br>None	
**7**	Support for attending meetings and/or travel	☐ **None**<br>None	
**8**	Patents planned, issued or pending	☐ **None**<br>None	
**9**	Participation on a Data Safety Monitoring Board or Advisory Board	☐ **None**<br>None	
**10**	Leadership	☐ **None**	

		**Name all entities with whom you have this relationship or indicate none (add rows as needed)**	**Specifications/Comments (e.g., if payments were made to you or to your institution)**
	or fiduciary role in other board, society, committee or advocacy group, paid or unpaid	None	
**11**	Stock or stock options	☐ **None**<br>Ionetix	
**12**	Receipt of equipment, materials, drugs, medical writing, gifts or other services	☐ **None**<br>None	
**13**	Other financial or non-financial interests	☐ **None**<br>Abbott Laboratories, Abbvie, Advanced Micro Devices, Amgen, Baxter International, Boston Scientific, Bristol-Myers Squibb, Cardinal Health, Cigna, Eli Lilly, GE Healthcare, Intel, Ionetix, Johnson and Johnson, Medtronic, Merck, nVidia, Oracle, Pfizer, Viatris, Zimmer Biomet	

Please place an "X" next to the following statement to indicate your agreement:

☒ X

ICMJE DISCLOSURE FORM

Date:<br>March 8, 2026	Click or tap to enter a date.
Your Name:<br>Ravi V. Shah	Click or tap here to enter text.
Manuscript Title:<br>Plasma Proteomic Signatures of Lipoprotein(a) and Cardiovascular Disease in Young Adults	Click or tap here to enter text.
Manuscript Number (if known):<br>204287-JCI-CRPH-1	Click or tap here to enter text.

In the interest of transparency, we ask you to disclose all relationships/activities/interests listed below that are related to the content of your manuscript. "Related" means any relation with for-profit or not-for-profit third parties whose interests may be affected by the content of the manuscript. Disclosure represents a commitment to transparency and does not necessarily indicate a bias. If you are in doubt about whether to list a relationship/activity/interest, it is preferable that you do so.

The author's relationships/activities/interests should be defined broadly. For example, if your manuscript pertains to the epidemiology of hypertension, you should declare all relationships with manufacturers of antihypertensive medication, even if that medication is not mentioned in the manuscript.

In item #1 below, report all support for the work reported in this manuscript without time limit. For all other items, the time frame for disclosure is the past 36 months.

		**Name all entities with whom you have this relationship or indicate none (add rows as needed)**	**Specifications/Comments (e.g., if payments were made to you or to your institution)**
**Time frame: Since the initial planning of the work**			
**1**	All support for the present manuscript (e.g., funding, provision of study materials, medical writing, article processing charges, etc.) **No time limit for this item.**	☐ **None**<br>NIDDK U01DK123013-03, NIA R01 AG059729, NHLBI R01 HL136685, AHA Strategically Focused Research Network	
			Click the tab key to add additional rows.
**Time frame: past 36 months**			
**2**	Grants or contracts from any entity (if not indicated in	☐ **None**<br>None	

		Name all entities with whom you have this relationship or indicate none (add rows as needed)	Specifications/Comments (e.g., if payments were made to you or to your institution)
	item #1 above).		
**3**	Royalties or licenses	☐ **None**<br>None	
**4**	Consulting fees	☐ **None**<br>None	
**5**	Payment or honoraria for lectures, presentations, speakers bureaus, manuscript writing or educational events	☐ **None**<br>None	
**6**	Payment for expert testimony	☐ **None**<br>Amgen, Cytokinetics	
**7**	Support for attending meetings and/or travel	☐ **None**<br>None	
**8**	Patents planned, issued or pending	☐ **None**<br>None	
**9**	Participation on a Data Safety Monitoring Board or Advisory Board	☐ **None**<br>None	
**10**	Leadership	☐ **None**	

		Name all entities with whom you have this relationship or indicate none (add rows as needed)	Specifications/Comments (e.g., if payments were made to you or to your institution)
	or fiduciary role in other board, society, committee or advocacy group, paid or unpaid	Patent for ex-RNAs signatures of cardiac remodeling; pending patent on proteomic signatures of fitness and lung and liver diseases	
**11**	Stock or stock options	☐ **None**<br>None	
**12**	Receipt of equipment, materials, drugs, medical writing, gifts or other services	☐ **None**<br>None	
**13**	Other financial or non-financial interests	☐ **None**<br>None	

Please place an "X" next to the following statement to indicate your agreement:

☒ X

ICMJE DISCLOSURE FORM

Date: March 8, 2026	Click or tap to enter a date.
Your Name: Robert S. Rosenson	Click or tap here to enter text.
Manuscript Title: Plasma Proteomic Signatures of Lipoprotein(a) and Cardiovascular Disease in Young Adults	Click or tap here to enter text.
Manuscript Number (if known): 204287-JCI-CRPH-1	Click or tap here to enter text.

In the interest of transparency, we ask you to disclose all relationships/activities/interests listed below that are related to the content of your manuscript. “Related” means any relation with for-profit or not-for-profit third parties whose interests may be affected by the content of the manuscript. Disclosure represents a commitment to transparency and does not necessarily indicate a bias. If you are in doubt about whether to list a relationship/activity/interest, it is preferable that you do so.

The author’s relationships/activities/interests should be defined broadly. For example, if your manuscript pertains to the epidemiology of hypertension, you should declare all relationships with manufacturers of antihypertensive medication, even if that medication is not mentioned in the manuscript.

In item #1 below, report all support for the work reported in this manuscript without time limit. For all other items, the time frame for disclosure is the past 36 months.

		Name all entities with whom you have this relationship or indicate none (add rows as needed)	Specifications/Comments (e.g., if payments were made to you or to your institution)
**Time frame: Since the initial planning of the work**			
**1**	All support for the present manuscript (e.g., funding, provision of study materials, medical writing, article processing charges, etc.) **No time limit for this item.**	☐ **None**<br>None	
			Click the tab key to add additional rows.
**Time frame: past 36 months**			
**2**	Grants or contracts from any entity (if not indicated in	☐ **None**<br>Amgen, Arrowhead, Eli Lilly, Merck, NIH, Novartis, Novo Nordisk, Regeneron, 89Bio	

		Name all entities with whom you have this relationship or indicate none (add rows as needed)	Specifications/Comments (e.g., if payments were made to you or to your institution)
	item #1 above).		
3	Royalties or licenses	☐ **None**<br>None	
4	Consulting fees	☐ **None**<br>None	
5	Payment or honoraria for lectures, presentations, speakers bureaus, manuscript writing or educational events	☐ **None**<br>Wolters Kluwer (UpToDate)	
6	Payment for expert testimony	☐ **None**<br>Amgen, Avilar, CRISPR Therapeutics, Eli Lilly, Lipigon, New Amsterdam, Novartis, Precision Biosciences, Regeneron, UltraGenyx, Verve Therapeutics	
7	Support for attending meetings and/or travel	☐ **None**<br>Meda Pharma	
8	Patents planned, issued or pending	☐ **None**<br>None	
9	Participation on a Data Safety Monitoring Board or Advisory Board	☐ **None**<br>None	
10	Leadership	☐ **None**	

		Name all entities with whom you have this relationship or indicate none (add rows as needed)	Specifications/Comments (e.g., if payments were made to you or to your institution)
	or fiduciary role in other board, society, committee or advocacy group, paid or unpaid	Methods and systems for biocellular marker detection (PCT/US2019/026364); Immunothrombotic conditions (PCT/US2021/63104926); Lp(a) vs non-Lp(a) apoB quantification (PCT/US2021/63248837)	
**11**	Stock or stock options	☐ **None**<br>None	
**12**	Receipt of equipment, materials, drugs, medical writing, gifts or other services	☐ **None**<br>None	
**13**	Other financial or non-financial interests	☐ **None**<br>MediMergent, LLC	

Please place an "X" next to the following statement to indicate your agreement:

☒ X
